# The protocol for mesoscopic wide-field optical imaging in mice: from zero to hero

**DOI:** 10.1093/biomethods/bpaf090

**Published:** 2025-12-12

**Authors:** Evgenia N Kislukhina, Natalia V Lizunova, Alexander M Surin, Zanda V Bakaeva

**Affiliations:** Laboratory of Neurobiology and Fundamentals of Brain Development, National Medical Research Center for Children’s Health Federal State Autonomous Institution of the Russian Federation Ministry of Health, Building 1, 2 Lomonosovsky Prospekt, Moscow, 119991, Russia; Laboratory of Neurobiology and Fundamentals of Brain Development, National Medical Research Center for Children’s Health Federal State Autonomous Institution of the Russian Federation Ministry of Health, Building 1, 2 Lomonosovsky Prospekt, Moscow, 119991, Russia; Laboratory of Fundamental and Applied Problems of Pain, Institute of General Pathology and Pathophysiology, 8 Baltiyskaya Street, Moscow, 125315, Russia; Laboratory of Neurobiology and Fundamentals of Brain Development, National Medical Research Center for Children’s Health Federal State Autonomous Institution of the Russian Federation Ministry of Health, Building 1, 2 Lomonosovsky Prospekt, Moscow, 119991, Russia; A.P. Nelyubin Institute of Pharmacy, I.M. Sechenov First Moscow State Medical University (Sechenov University), Building 2, 8 Trubetskaya Street, Moscow, 119991, Russia

**Keywords:** cranial window, skull thinning, calcium imaging, haemodynamics, intrinsic brain signals

## Abstract

This article provides protocols that enable researchers to master mesoscopic wide-field optical brain imaging from scratch. The protocols describe surgery for wide-field cranial window creation in mice, as well as the imaging process and setup. The protocols for components of the imaging system selection and assembly, creation of a headplate for fixation, and training mice are also provided. The final section briefly outlines methods for data processing. The described procedure can be used to visualize the dorsal cortex using wide-field optical imaging and laser-speckle contrast imaging methods. The distinguishing features of our protocol include: a wide cranial window (up to 60% of the entire cortex), skull thinning (without craniotomy), a UV-curable transparent coating (gel polish), and the ability to perform measurements in awake, behaving mice. During the surgery, a helicopter-shaped headplate with a lower surface congruent to the skull surface is mounted on the mouse’s head. This lightweight headplate allows for secure head fixation during movement eliminating the need for alignment during data analysis. Cranial window remains sufficiently transparent for at least three months. Wide-field optical imaging enables the recording of brain haemodynamics and energy metabolism (FAD concentration dynamics) in wild-type mice. The use of transgenic animals expressing genetically encoded sensors allows for the measurement of ions concentrations (e.g. Ca^2+^-dynamics) and other compounds (e.g. glutamate). This article describes the simultaneous measurement of changes in oxy-, deoxy-, and total haemoglobin concentrations in combination with various intracellular parameters: Δ[FAD], Δ[Ca^2+^], or ΔpH with Δ[Cl^-^].

## Introduction

*In vivo* imaging techniques are rapidly advancing in biological research, offering significant advantages over traditional histological and biochemical methods. These techniques allow for chronic studies within the same animal, reducing the number of subjects required and enhancing data quality. Notably, *in vivo* brain imaging can now be performed in awake animals, eliminating anaesthesia-related confounds [1].

Among the neuroimaging techniques, the following can be highlighted: traditional methods, used in both experimental and clinical contexts, including structural and functional MRI, computed tomography, electroencephalography, electrocorticography, implanted electrodes, and ultrasound (Doppler) imaging [2–4]. However, optical methods have gained prominence in experimental neuroscience, including two- and three-photon microscopy, miniscopy, Raman microspectroscopy, laser speckle contrast imaging (LSCI), and wide-field optical imaging (WFOI) [5, 6]. A recent achievement is the clinical application of optical techniques: functional near-infrared spectroscopy (fNIRS) [7] and Raman microspectroscopy [8]. WFOI, employing a cortex-wide cranial window, enables high spatial and temporal resolution imaging but is limited by light scattering, restricting depth measurements to the cerebral cortex [9].

Brain activity can be observed due to the optical properties of haemoglobin (this mode is referred as intrinsic optical signal imaging, IOSI/ISI) [10, 11], FAD [12] and artificially created dyes [13] or biosensors [14]. An alternative optical approach exploits the specific scattering properties of a laser beam, enabling spectroscopy [15] or blood flow velocity measurements [16]. The excitation of neurons causes vasodilation and an increase in local blood flow by the mechanism of neurovascular coupling [17]. This is accompanied by an increase in total (HbT) and oxygenated haemoglobin (HbO), and a decrease in deoxyhaemoglobin (HHb [18]); the last often referred to as HbR, although the term ‘reduced’ is biochemically misleading [19]. In addition, electrical activity requires the consumption of energy substrates (e.g. reduced flavine adenine dinucleotide), which is followed by an increase in the ratio [FAD]/[FADH_2_] [20]. Changes in FAD and haemoglobin concentrations can be measured photometrically, since haemoglobin is a pigment and absorbs light in the visible part of the spectrum (with distinct absorption spectra for HbO and HHb), and FAD (but not FADH_2_) has significant fluorescent properties [12, 21]. The use of genetically encoded fluorescent sensors allows researchers to expand the panel of visualized processes. Among all genetically encoded sensors, the most widely used is the Ca^2+^ sensor GCaMP [22, 23], however, there are also sensors for pH, Cl^-^, glutamate, etc [24–28]. An increase in the concentration of a particular ion or molecule causes an increase in fluorescence or quenching of the sensor. For such experiments, transgenic mouse lines are used, or viral transduction is carried out to express protein sensors in brain cells [22, 26, 29–32]. All optical neuroimaging methods require the creation of a cranial window to enable optical access to the brain, which is achieved in various ways.

**Figure 1:**
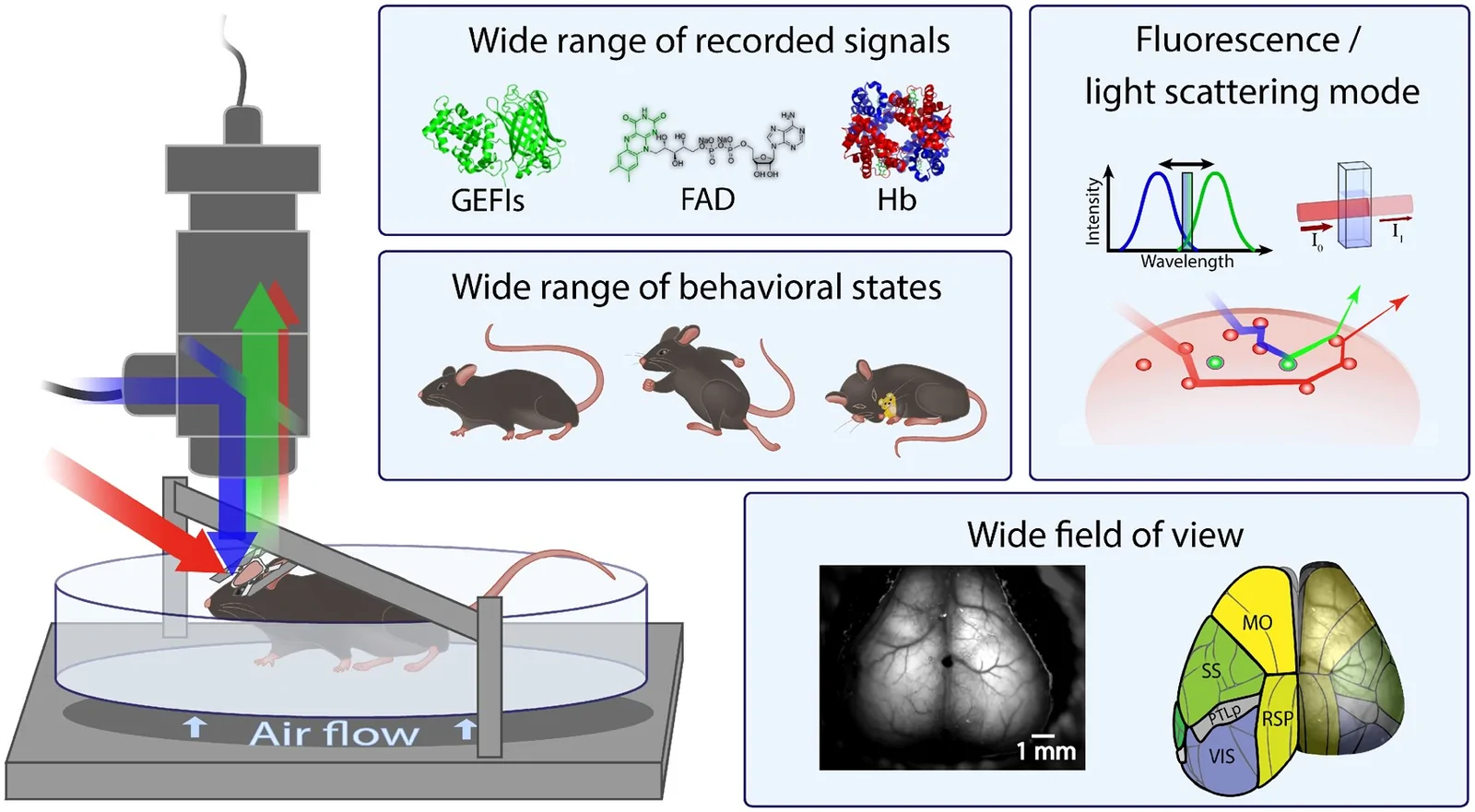
The main advantages of wide-field optical imaging. Wide-field optical imaging enables the measurement of changes in the concentrations of endogenous fluorophores (e.g. FAD) and pigments (e.g. haemoglobin, Hb), as well as signals from artificial genetically encoded fluorescent indicators (GEFIs), in both awake freely moving animals and anaesthetized ones. Measurements can be performed in fluorescence or light-scattering (reflectance, intrinsic optical signal) modes. A wide cranial window enables visualization of all major cortical regions except the auditory cortex. MO—Somatomotor areas; PTLp—Posterior parietal association areas; RSP—retrosplenial area; SS—Somatosensory areas; VIS—visual areas, according to the Allen mouse brain common coordinate framework [52]

One advantage of wide-field optical neuroimaging is its ability to capture signals from a large area of a dorsal part of the cerebral cortex (dorsal cortex) simultaneously (Fig. 1). While signal detection through an intact skull is possible, it is affected by diploic vessel activity and significant light scattering. To overcome these issues, researchers either perform a craniotomy or thin the skull [21, 33–37]. The latter is a less invasive approach but requires specific skills to achieve optimal results.

Another key advantage is the ability to record brain activity in awake animals. This requires the development of a system that ensures stable head fixation while allowing for the simulation of natural locomotion [5, 38–45].

Additionally, wide-field optical neuroimaging offers remarkable versatility. By integrating different LEDs (light-emitting diodes), stimulus delivery devices, and genetically encoded sensors, researchers can address a wide range of experimental questions [21, 46–51]. While commercial imaging systems are available, many laboratories prefer to assemble custom setups using various components to better suit their specific research needs.

Our protocols provide a step-by-step guide to cranial window preparation by skull thinning with a transparent protective coating, imaging system assembly, and animal training for imaging sessions. By outlining a cost-effective and adaptable approach to WFOI, we aim to make high-precision neural imaging more accessible to researchers. The protocols were extensively tested and refined. Selected results have been published in [53].

## Materials and methods

The equipment, tools, and consumables used in this study are listed in Supplementary Table S1.

### Headplate design

**Figure 2:**
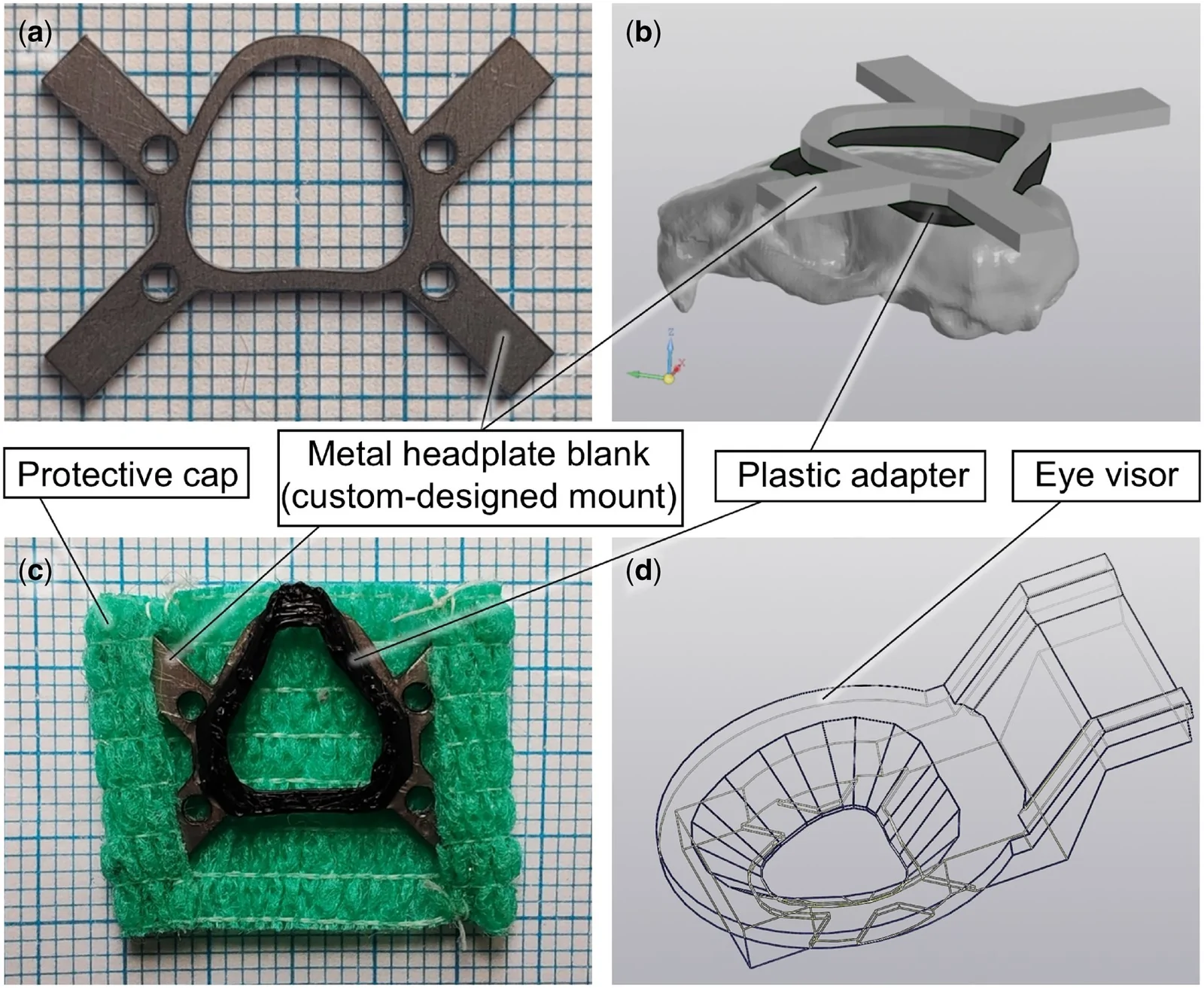
A headplate for securing an animal’s head under a microscope. (**a**) A titanium headplate blank (custom-designed mount); (**b**) a 3D model of an adapter that is congruent with the surface of the mouse’s skull; (**c**) the whole headplate: metal blank with a glued plastic adapter and a removable protective cap (ventral view); (**d**) a 3D model of a visor, which prevents eye stimulation from LEDs of the illumination system. Panels (b) and (c) are edited screenshots obtained from the KOMPAS-3D software (ASCON, Russia)

This section describes the process of manufacturing headplates (Fig. 2) that are compatible with the Mobile Homecage^®^ system (Neurotar, Finland). This headplate design has the following advantages: wide field of view; lightness and compactness; congruence to the surface of the skull; secure head fixation during imaging, preventing frame alignment issues during data processing.

The protocol describes headplate manufacturing from two parts: a metal blank and a concave adapter made of plastic. At the end of the protocol, a method for manufacturing a protective cap for the brain surface, which prevents injury, is described. The Supplementary File S2 provides alternative protocols for headplate design.

#### Manufacturing headplates

1. Order laser-cut flat reusable headplate blanks from a 1 mm thick sheet of steel or titanium according to the drawing (see Supplementary File S3).
*Note: Titanium is preferred because it is lighter and does not corrode.*
2. Print the black PET-G plastic headplate adapters using a 3D printer (for the 3D model, please see Supplementary File S3). If needed, refine them with a needle file.
*Note: The black colour of the plastic prevents light from penetrating through the headplate.*
3. Degrease the surfaces. Apply cyanoacrylate glue to the metal workpiece. Place the plastic headplate adapters on top to match contours of the two items. Press firmly for 10 s and allow to dry for 1–2 min (Fig. 2).
*Note: If these headplates do not meet your requirements, please see Supplementary File S2 for alternative protocols, including the creation of custom adapters from epoxy resin*.
4. 3D-print an eye visor using black PET-G plastic (for the 3D model, please see Supplementary File S3).
5. Cut a 20 × 32 mm piece of veterinary bandage with bitter impregnation. Fold 4 mm on each side to make a 20 × 24 mm piece. Sew it up to make two pockets for the ‘propeller blades’ of the helicopter-like headplate (Fig. 2C). Test whether it is easy to put it on and off. The cap should not touch the mouse’s eyes.
*Note: The protective cap prevents brain damage and dust ingress. The cap is essential for keeping male mice together, as they are prone to fighting. It is not strictly necessary for solitary confinement or for keeping females together.*

#### Reuse of metal blanks

6. Remove the headplate from the mouse’s head at the end of an experiment. Place the headplates in acetonitrile for a minimum of 2 h to dissolve dental resin and glue.
7. Remove any remaining glue and plastic with sandpaper or a needle file.

### Wide-field thinned cranium surgery

**Figure 3:**
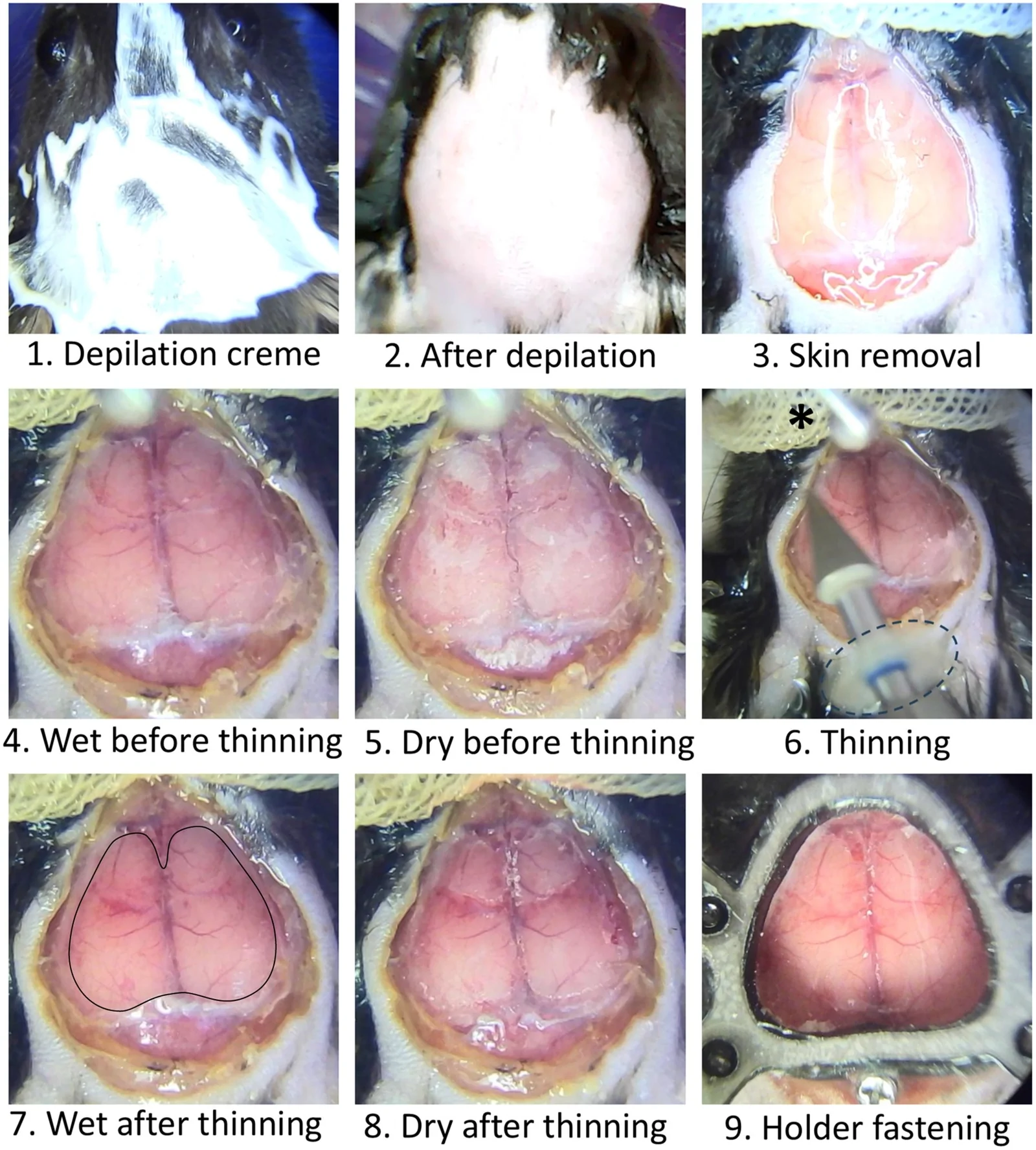
Stages of the surgery. (1) Apply depilatory cream for 1 min. (2) Remove depilatory cream with a nail stick and a damp cotton swab. (3) Sterilize surgical field; remove skin and fascia. (4) Set up continuous rinsing with saline using a peristaltic pump; use gauze for drainage. (5) Check skull thickness periodically by drying with an air flow. (6) Thin the skull using a microdrill with progressively softer drill bits; the asterisk indicates the tip of the saline needle and drainage gauze, cotton wool is placed underneath the head for saline absorbance; the dashed line denotes the protective ‘skirt’ for the drill bit to prevent fluid from entering the drill. (7) The wet thinned skull has minimal differences from the intact one; the solid line denotes the thinned area. (8) Good visibility of blood vessels trough a dry skull indicates proper thinning. (9) Secure the screw into the occipital bone; fasten the holder with cyanoacrylate glue and dental resin; cover the skull with cyanoacrylate glue, base and top gel polish

This section describes a surgical procedure to create a wide cranial window (Fig. 3). The main advantages of this protocol over craniotomy include: lower invasiveness, as the cranial cavity remains intact; low risk of bleeding; and ease of coating. The main limitations include bone regrowth if the osteogenic layer is not removed completely, and the requirement for a delicate surgical technique as a neuroinflammatory response may occur. For a video protocol see https://www.youtube.com/@WIFIOPIA. Detailed techniques and material specifications are available in Supplementary Table S1 and Supplementary File S2. A troubleshooting guide is in Supplementary File S4.

All experiments with animals should be conducted in accordance with local ethical guidelines.

#### Preparation for surgery. Perform one day or more before surgery

1. Cover all work surfaces and equipment with plastic film, leaving the tips of ear bars, the fronts of the dental clamp, the binocular lenses, the light source, and the tip of the saline needle (see item 7 in the section ‘Preparation for surgery. Perform one day or more before surgery’) uncovered.
2. Prepare protective ‘skirts’ for drill bits by cutting approximately 7 mm plastic discs with central holes matching the cutter shaft. Secure them approximately 5 mm from the tip using gel polish.
*Note: Steps 1–2 prevent fluid from entering the drill and other equipment.*
3. Prepare the following solutions aseptically (Table 1): prophylactic antibiotic Marbofloxacin (0.00017 mg/ml in water for injection), analgesic Ketoprofen (1.65 mg/ml in saline), and Dexamethasone (0.35 mg/ml in saline). Store at +4 °C in aliquots. Dexamethasone was used to prevent neural oedema [54]. Different protocols of pre- and post-operative pharmacological treatment are used for cranial window preparation; we recommend following the guidelines of the local ethical committee and veterinary service [54–56].
4. Prepare angled cannulae tips 18G, 45°; bend 27G needles to an L-shape for skull cleaning, glue application, bubble removal, and to a gentle C-shape (about a quarter circle) for lacquer marking.
5. Create a visor from aluminium foil to protect the mouse’s eyes from UV light.
6. Cut gauze into 2 × 6 cm strips and prepare cotton swabs and cotton wool for cleaning and drainage; store small supplies in one place for convenience.
7. Route the silicone tubing from the peristaltic and air pumps to the stereotaxic frame above, attaching appropriate tips or needles for fluid delivery and airflow control.

**Table 1.** Medications.

Reagent	Final concentration	Amount
**Marbofloxacin 0.00017 mg/ml solution**		
Marbobel 2 (Marbofloxacin 20 mg/ml)	8.3%	1 ml
Water for injection	91.7%	11 ml
**Ketoprofen 1.65 mg/ml solution**		
Ketonal (Ketoprofen 50 mg/ml)	3.3%	0.5 ml
Saline (0.9% NaCl)	96.7%	14.5 ml
**Dexamethasone 0.35 mg/ml solution**		
Dexamethasone for injection (Dexamethasone 4 mg/ml)	8.8%	132 µl
Saline (0.9% NaCl)	91.2%	1368 µl

*Notes:*

*– Immediately dry and rinse equipment with distilled water if contact with saline occurs, to prevent corrosion.*

*– Marbofloxacin may precipitate in saline; use water for dilution.*

#### Surgery

##### Preparation for surgery. Perform on the day of the operation

1. Ensure the availability of all tools, materials, and medications.
2. Disinfect the workspace, instruments, and screws (avoid heating headplates: each plate carries a bonded plastic adapter and adhesive that are not heat-stable; they can be treated briefly with 70% ethanol prior to gluing).
3. Prepare materials for cleaning the depilatory cream (distilled water, nail stick, cotton swab) and a clean cage with food and water.
4. Flush the perfusion pump tubing with 50 ml 6% peroxide, 50 ml distilled water, and 25 ml saline, unless reused consecutively.
5. Set up the perfusion pump by hanging a 200 ml saline infusion bag, and connect it to the pump tubing. Configure the flow rate to 1–2 ml/min.
6. Turn on the temperature-controlled pad (set to 37 °C).

##### Animal preparation

7. Anaesthetize the animal in the induction chamber of the isoflurane system (4% isoflurane, 96% air, 1 L/min flow rate).
8. After anaesthesia induction, quickly shave the surgical area using an electric shaver.
9. Transfer the animal to the stereotaxic frame. When securing the animal, gently move the mouse’s tongue to one side to prevent airway obstruction during prolonged anaesthesia. Reduce isoflurane to 1%–2.5% to maintain stable anaesthesia. Check the flow rate (1 L/min) every 20 min.
10. Apply eye gel regularly (∼every 15 min) to prevent drying.
11. Administer dexamethasone intramuscularly (0.7 mg/kg, V = 2 µl/g mouse) into the anterolateral quadriceps using a 31 G needle; split the total volume into two equal aliquots and inject bilaterally (two sites). The aqueous dexamethasone sodium phosphate formulation was used, which yields rapid i.m. peaks, so dosing at induction aligns peak exposure with the early, more tissue-reactive steps [57, 58].
12. Insert and secure a lubricated rectal probe.
13. Inject 0.1 ml of 2% lidocaine subcutaneously under the scalp.
14. Place cotton wool under the head to absorb saline.
15. Adjust the microscope and lighting for binocular viewing.

##### Preparation of the surgical field

16. Apply depilatory cream to the scalp for 1 min, covering a slightly wider area than the planned incision; remove the cream and fur using a nail stick, then clean the skin with a damp cotton swab (Fig. 3, panels 1 and 2).
*Note: Exposure time may be individually adjusted for the specific cream lot/formulation, but prolonged contact must be avoided to prevent skin injury.*
17. Disinfect gloves and clean the mouse’s head three times with povidone–iodine antiseptic, followed by three wipes with 70% ethanol to ensure that no povidone–iodine residue remains, as it may cause skin irritation.
*Note: Avoid contact of ethanol and depilatory cream with the eyes.*
18. Confirm the absence of a pedal reflex. Check the reflex every 15 min.
19. Excise the scalp to expose the occipital, parietal, frontal, and part of the nasal bones; do not expose muscles; ensure smooth incision edges (Fig. 3, panel 3).
20. Control capillary bleeding by applying a topical haemostatic agent for 1-2 min.
21. Remove all connective tissue from the skull surface, including the edges, to ensure proper headplate fixation and prevent interference with drilling.

##### Thinning of the skull

22. Place saline-soaked gauze over the nasal bones, covering the eyes and retracting the vibrissae.
23. Ensure stable head fixation in the stereotaxic frame: gently test the skull for movement (from side to side) with forceps under the microscope. Reinforce the ear bar placement if needed.
24. Activate the peristaltic pump (1–2 ml/min) and adjust the saline needle to ensure that a saline drop lands rostral to the thinning site.
25. Set the microdrill speed (∼25 000 rpm) and insert the medium-grit diamond drill bit.
26. Place the index and middle fingers of the non-dominant hand on the ear bars. Stabilize your working hand by resting the little finger and ulnar side on top of the index finger or on the heating pad, then begin skull thinning with the microdrill.
27. First, thin the frontal bones; control bleeding if needed (Fig. 3, panel 6). Maintain a tangential motion for uniform thinning.
28. Slightly thin the sutures, avoiding over-thinning near the rostral quarter of the superior sagittal sinus (Fig. 3, panel 7). Keep the drill parallel to the skull surface and thin across both bones to prevent bone edges from pressing into the brain.
29. Stop the drill and pump; dry the skull with airflow and assess the degree of thinning. Adequate thinning is indicated by: pinkish colour and visibility of small vessels, with the colour and transparency remaining unchanged during drying (Fig. 3, panels 4–8). The thinned surface may deform under minimal pressure, which can cause brain injury.
30. Activate the peristaltic pump, keeping in mind the position of the thinned areas. Resume thinning of the parietal bones with continuous movements across the sagittal suture. Take care around the thinned regions, as their boundaries are fragile. Maintaining uniform thinning throughout the process is crucial to prevent cracking.
31. Refine thinning of the sutures with medium-grit silicone-diamond polisher (blue). Pay special attention to the boundaries of the frontal bones as they are prone to regrowth. Polish the surface using progressively softer cutters: medium-grit silicone-diamond polisher (blue), fine-grit (red), then extra-fine grit (grey). Leave the inner bone layer intact.
32. Continue until the skull is uniformly transparent when both wet and dry, with visible vasculature.

*Notes:*

*– Stable head fixation and stabilization of the drilling hand reduce the risk of brain injury.*

*– Proper drainage prevents mouse aspiration and keeps optics clean.*

*– Use drill bits with the base diameter 4-5 mm, 7-8 mm length, bullet or conical shape.*

*– Bleeding may occur from diploic vessels, this is expected.*

*– Uneven thinning increases the risk of fracture.*

*– Avoid drying the fully thinned skull for extended periods.*

##### Application of coating and headplate fixation

33. Reapply eye gel and dry the skull (Fig. 3, panel 8). Use the airflow to gently adhere the skin to the skull surface.
34. Apply a thin layer of liquid cyanoacrylate glue to the thinned area using the L-shaped 27G needle. Glue the skin edges to the skull to prevent infection. Allow it to harden.
35. Confirm head stability (see item 23 in the section ‘Surgery’) and insert a M1 screw into an indentation pre-drilled with a tungsten carbide drill bit in the occipital bone, without penetrating fully.
36. Use a No15 scalpel blade to score nasal bones for proper headplate fixation.
37. Sterilize and test-fit the headplate, then glue it onto the clean, dry skull surface with gentle pressure. Do not glue the headplate to skin or muscles to prevent instability.
38. Shield the eyes with the foil visor. Apply a layer of base coat gel polish to the thinned skull. Cure under UV light for 15s, then take a break for 10s (to prevent thermal damage from exothermic polymerization), then cure under UV for 60s. Repeat the same procedure with the top coat.
39. Mark Bregma with black nail polish if no longer visible. Use a C-shaped needle.
40. Prepare self-curing acrylic dental resin: mix ∼0.2 g of powder and 0.25 ml of liquid in 5 ml silicone mixing cup. Apply it gradually around the headplate using a syringe fitted with 18G angled cannulae tips, sealing the entire perimeter including skin edges, but avoiding contact with the cranial window or eyes (Fig. 3, panel 9).
41. After the resin sets, remove the animal from the frame. Administer marbofloxacin (8 mg/kg, V = 5 µl/g mouse), ketoprofen (8.5 mg/kg, V = 5 µl/g mouse), and 0.5 ml saline subcutaneously.
*Note: In our study, we used this postoperative regimen. However, to align onset of action with the surgical stimulus and ensure pre-emptive analgesia and pre-emptive antibiotic prophylaxis, we recommend administering antibiotic and ketoprofen at induction (prior to skin incision) so that by the time of the skin incision (∼40 min) and skull thinning (∼60 min) these agents have reached effective levels* [42, 55, 59].
42. Discontinue isoflurane and place the mouse in a clean, heated single-housing cage. Monitor for at least 1 h after the operation.
43. For the next 3 days, administer marbofloxacin (8 mg/kg, V = 5 µl/g mouse), ketoprofen (8.5 mg/kg, V = 5 µl/g mouse) subcutaneously daily while monitoring recovery and inflammation.
44. Continue rehabilitation for at least 14 days (preferably up to 1 month) before experiments.

*Notes:*

*– Cyanoacrylate glue eliminates the osteoblast population* [60].

*– Prevent bubble formation and blood ingress in the glue, gel polish, and resin.*

*– If possible, make a flat surface with gel polish, glare may occur on a spherical surface.*

*– Avoid excessive resin on the sides of the headplate to ensure proper holder mounting afterwards.*

*– If the semi-transparency or autofluorescence or of the dental resin affects imaging, use a black nail polish to paint over the outside or use a dental cement instead.*

*– Post-operative injections (antibiotic and analgesic) should be administered subcutaneously in the back area rather than into the scruff to avoid disturbing the surgical site.*

*– According to Regulation (EU) 2025/877, certain substances previously used in UV/LED gel polishes, including Trimethylbenzoyl Diphenylphosphine Oxide (TPO), have been banned in the European Union. The gel polishes used in this study may contain components that are now restricted. TPO is classified as a potential carcinogenic, mutagenic, or reproductive toxic (CMR) substance, and gel polishes often contain irritant acrylate or methacrylate monomers that may cause skin or respiratory irritation. Therefore, it is recommended to use TPO-free formulations in future work and to handle all materials with gloves and a protective mask in a well-ventilated area (preferably under a fume hood).*

For more detailed description and practical details, please see Supplementary Table S1, Supplementary Files S2, S4, and the video-protocol.

### Component selection and assembly for wide-field optical imaging system

This section describes the assembly and configuration of the WFOI system. The first part provides the minimal required configuration for the measurement of changes in HHb or HbT only in an anaesthetized animal. The second part describes further modifications to enable the measurement of multiple parameters–HbO, HHb, and HbT, alongside FAD (in wild-type mice), or Ca^2+^ (in mice expressing GCaMP), or pH and Cl^-^ (in mice expressing ClopHensor)—as well as sensory stimulation in awake, behaving animals.

The system can be easily modified for laser speckle contrast imaging (using NIR laser illumination, e.g. 780–850 nm) or adapted for different fluorescent sensors. The limitation for fluorescent excitation is the short-wavelength range (≤430 nm), as it penetrates poorly through the skull coating.

**Figure 4:**
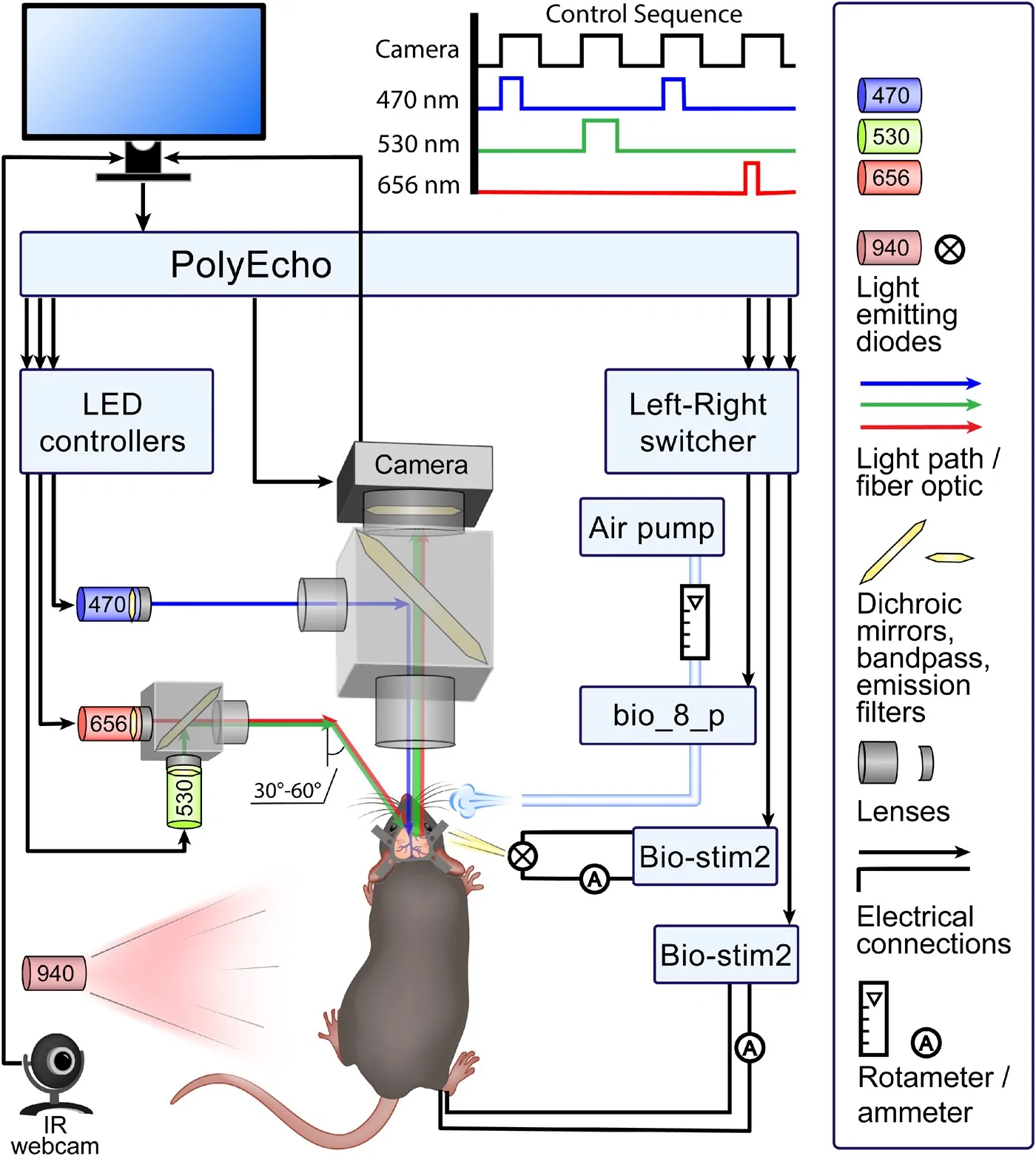
Schematic diagram of our WFOI system, with the illumination setup on the left and sensory stimulation devices on the right. An IR webcam with a 940 nm LED records mouse behaviour

The fundamental design of the imaging system is shown in Fig. 4. Since individual components used to build imaging systems may vary across laboratories, we have focused on general principles of component selection and assembly, rather than the specific details of our setup.

#### The minimum configuration

The minimum configuration for a WFOI system includes:

- one LED for haemoglobin imaging;
- an LED controller to regulate current;
- a lens;
- a camera for image acquisition;
- an LED stand;
- a camera stand;
- two tables;
- a computer;
- software for controlling the camera and the LED controller;
- a multi-channel controller to synchronize the LED controller, the camera, and the computer;
- a head-fixation frame;
- a heating pad for the mouse.

This setup is enough for measuring Δ[HbT] or Δ[HHb] (depending on the chosen LED wavelength; see Table 2) in an anaesthetized animal.

**Table 2.** Decision-making for LED wavelength choice.

Measured molecules	LED’s central wavelength	Reason
HbT	505 or 530 or 540 or 560 or 590	Isosbestic points for HbO and HHb in the green-red spectrum are at 500, 530, 545, 570, and 584 nm Use a narrow bandpass filter
HHb	625-680	HHb has a 10× higher molar extinction coefficient than HbO in the red spectrum. Between 625 nm and 680 nm, HHb’s extinction decreases significantly
HbT, HbO, HHb	590 and 625 nm	Longer wavelengths penetrate deeper into tissue, so yellow and red LEDs will sample similar depths [61]. Theoretically, any two wavelengths are suitable. For optimal accuracy, one should be isosbestic, and the other should be in the red region
HbT, HbO, HHb, GCaMP	530 and 625 nm for Hb 470 nm for GCaMP	530 nm is near the emission maximum of GCaMP. Hb imaging is necessary for the correction of fluorescence signals [61] 405 nm may be used as a Ca-independent signal of GCaMP (not necessary) [49]
HbT, HbO, HHb, FAD	530 and 625 nm for Hb 470 nm for FAD	See above
HbT, GCaMP or FAD	530 for HbT 470 nm for GCaMP/FAD	One wavelength near the emission maximum is enough for correction using the regression method [66]
HbT, HbO, HHb, ClopHensor	530 and 625 nm for Hb 460, 505 and 560 nm for ClopHensor	See above
HbT, HbO, HHb, when intravenous dye is used (e.g. Rose Bengal)	E.g. 590 and 625 nm if Rose Bengal	Wavelengths outside dye absorption spectra [51]

The preferred wavelengths are highlighted with an underscore.

Optional components that can enhance the system include: an uninterruptible power supply (UPS), аn actively vibration-isolated table, a bandpass filter, collimating and focusing lenses, a light diffuser, and an external hard drive. However optional, antivibration table and UPS are strongly recommended.

In our setup, the core system consists of a collimated LED 530 nm for HbT (BLS-LCS-0530-03-22, Mightex, CA, USA); a 530 nm bandpass filter (ZET532/10x, Chroma Technology Corp., VT, USA); a BioLED light source control module (BLS-1000-2, Mightex, CA, USA); lenses (OBJ-MAO-020-LWD, MAO-1-25-G-1C, Mightex, CA, USA); a high-sensitivity camera (CXE-B013-U, Mightex, CA, USA); a rectangular U-shaped LED mounting stand (Thorlabs, NJ, USA); an XYZ-motorized camera system (Zaber, Canada; Mightex, CA, USA), a U-shaped stand for motorized system (Thorlabs, NJ, USA; Micro Control Instruments Ltd, England); a custom-made anti-vibration table 75 × 270 cm and a secondary table for a mouse with a keyboard; a Back-UPS (ES 700VA, APS, USA); PolyScan4 and BuffCCD Cam software (Mightex, CA, USA); a Legion T5 26IOB6 computer (Lenovo Group Limited, China), and a GeForce 3060 Ti GPU (NVIDIA, USA); a 6 TB WDBWLG0040HBK-XB external drive (Western Digital, Thailand); a 12-channel PolyEcho Intelligent I/O Control Module BLS-IO12-U (Mightex, CA, USA); a Mobile HomeCage^®^ (Neurotar, Finland), and a ThermoStar Homeothermic Monitoring System (RWD, China). The detailed description of mounting stands and computer characteristics are provided in Supplementary Table S1.

*Note: Based on our experience, the camera, BuffCCD Cam software, the anti-vibration table, and the UPS included in the system are not optimal and may benefit from replacement (see steps 6, 3, 2, and 3 in sections ‘Optical system’, ‘Control system’, ‘Room, electrical supply and stands’, ‘Room, electrical supply and stands’, respectively, and Supplementary Table S1 for recommended options) The equipment does not necessarily have to be expensive (see the Discussion section), but a high-sensitivity camera is crucial for achieving a good signal-to-noise ratio.*

It is worth noting that, in WFOI/fMRI literature HbR is often used for deoxyhaemoglobin [61–63]. Because ‘reduced’ is biochemically misleading (deoxyhaemoglobin is not reduced in the classical electrochemical sense [19]), we use HHb instead [18, 64], which also underscores its role in pH buffering via proton binding.

##### Room, electrical supply and stands

1. Choose a dedicated recording room. Light, noise, and vibrations can interfere with imaging, therefore, cover windows with roller shutters.
*Note: Temperature control in the room is important to prevent LED overheating.*
2. Set up a table for the LED, the camera and the head-fixation frame. An actively vibration-isolated table is recommended. Set up another table for the devices, that can cause vibrations (such as the computer).
*Note: Ensure that monitor light does not reach the experimental setup. Place the monitor behind the mouse to avoid unintended visual stimulation.*
3. All critical equipment should be backed up with a UPS of appropriate VA capacity (an online UPS is optimal). Connect the UPS (or multiple units if required) to outlets on separate electrical circuits.
4. Install the head fixation stand (e.g. a stereotaxic frame). Assemble the LED mounting stand and the camera stand.
*Note: At least one of the following should be movable to allow proper focusing: the camera stand or the head frame. Tighten all screws securely to minimize vibrations. For further vibration control, consider fixing the stands to the tabletop. If the frame shifts by more than a pixel, post-recording alignment will be required (see Section ‘Image annotation and brain masking’).*

##### Optical system

**Figure 5:**
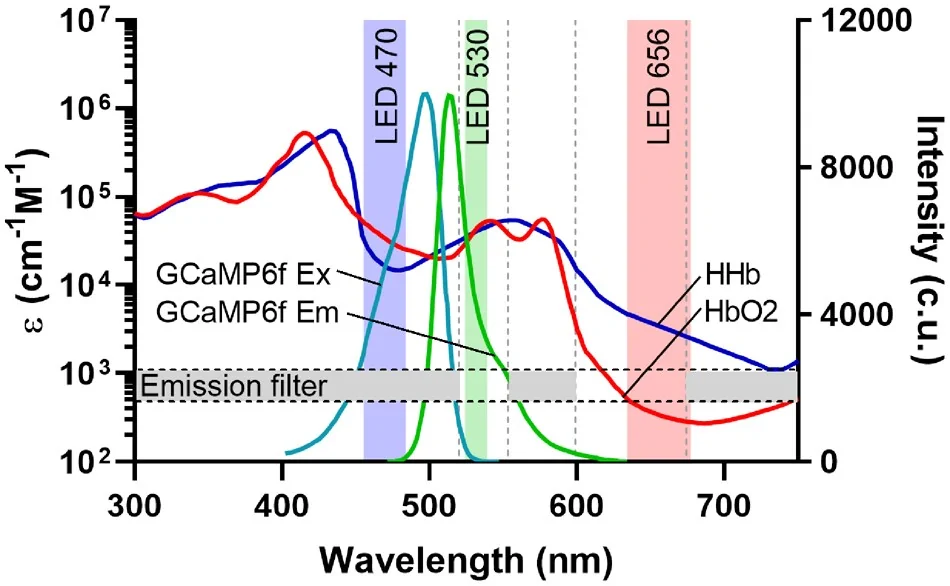
Optical spectra of our WFOI setup for the measurement of oxyhaemoglobin (HbO) and deoxyhaemoglobin (HHb) alongside Δ[Ca^2+^]_i_. The figure shows the molar extinction coefficients for HbO and HHb, and the excitation and emission spectra of GCaMP6f. LED spectra are shown after bandpass filers (ET470/24m, ZET532/10x, AT655/30m). The white sectors on the ‘Emission filter’ stripe correspond to the transparency windows of the dichroic mirror (59009bs) and emission filter 59026 m (all filters by Chroma Technology Corp., VT, USA). The same configuration can be used for Δ[FAD] measurements in wild-type mice (not shown)

1. Assemble the LED for light scattering on its stand to provide lighting at an angle of 30°–60°. We recommend LED models with passive cooling and a central wavelength of 530 or 590 nm for HbT and 625 nm for HHb (Table 2). The use of one LED will not allow precise measurements as HbO and HHb spectra overlap (Fig. 5). To measure the Δ[HbT] and Δ[HHb] precisely see item 1 in section ‘HbO, HHb and HbT measurements’ and section ‘Scattering signal conversion to haemoglobin concentrations’.
*Note: Lighting at a 90° angle for scattering creates glare from the skull surface, interfering with brain activity observations.*
2. Add a bandpass filter of ∼20 nm bandwidth after the LED to improve measurement accuracy. The recommended LED output power is 200–500 mW when a narrow bandpass filter is used.
*Note: Lower-power LEDs or optical filters with narrower bandwidths may require longer exposure times (10–20 ms)—ensure this is acceptable for your experiment.*
3. Mount collimating lenses to direct LED light. This reduces light loss.
*Note: LED light may act as an unintended visual stimulus for the mouse.*
4. Connect the LED to the LED controller. The controllers must automatically trigger the LED and manage the electrical current (not voltage) supplied to the LED to regulate brightness. Always consider the maximum allowable current for the LED.
*Note: Use LEDs and LED controllers from the same manufacturer.*
5. Set up a light diffuser between the LED and the mouse. You can make it from glass or PET plastic sanded with sandpaper. Another option is to use a circular polarizing filter mounted on the lens, but it reduces the overall image brightness by 75% or more.
*Note: A diffuser helps to eliminate glare. Surface irregularities of the skull become more noticeable, but this does not hinder the recorded brain activity. Another solution is to apply glycerol and a coverslip to the cranial window to match the refractive index, but we recommend to create flat surface with gel polish during surgery.*
6. Select a camera. We recommend using a monochrome camera with external trigger control (TTL signal), a frame rate of 100 Hz at a resolution of at least 256 × 256 pixels, and low dark current. However, a maximum frame rate of 10 Hz is sufficient for HHb- or HbT-only measurement. (If the camera’s dark signal is not zero, increase the frame rate to capture dark frames; if sensitivity is low, increase the frame rate for subsequent temporal binning.) The captured images should have a bit depth of at least 12 bits; preferably 16 or 32 bits.
*Note: 8-bit images can also be used, but the processing quality will be lower. Resolution is not critical, as for WFOI analysis binning down to 64 × 64 pixels can be used, provided that the experimental objectives allow such resolution. A camera with global shutter is preferable.*
7. Attach the camera to the lens and set it up on the camera stand above the animal’s head. A 2× – 4× lens with a working distance of 10 cm is recommended.

*Notes:*

- *A lens with a shorter working distance will capture more signal but will make mouse manipulation more difficult.*
- *Although optional, steps 2,3, and 5 markedly enhance imaging quality.*

##### Control system

1. Connect the LED controllers to an analogue output of a multi-channel controller. Connect the camera TTL trigger to a digital output. The multi-channel controller will transmit signals from the computer. The number of required channels depends on the number of wavelengths used–one per wavelength, one for the camera, and one per external device (e.g. a stimulator).
2. Connect all devices to a computer or laptop. The computer should meet the following minimum system requirements: 16 GB of RAM, 512 GB of storage (preferably SSD), an Intel Core i5 or equivalent processor, and Windows 10/11 (64-bit), USB 3.0 support (at least four ports).
*Note: For optimal performance, we recommend a computer with 64 GB of RAM, an Intel Core i7 (or equivalent) processor, and a dedicated graphics card (GPU). Pay attention to the number of USB ports: in a full setup (see the following sections), eight ports will be occupied.*
3. Install all necessary software for controlling the LEDs and the camera. This can be commercial software provided with the multi-channel controller and the camera or a third-party software (e.g. Micro-Manager 1.4 for the camera [65]). The software should be capable of starting and stopping recordings, controlling the activation, intensity, and duration of LED illumination, adjusting camera exposure time, synchronizing frame exposure with LED activation, and configuring image-saving parameters (binning, gain, file format, automatic file naming, save path). Additionally, it should allow pre-recording adjustments, including a preview mode for camera focusing and LED duration selection.
4. For raw data storage, select an external hard drive (6–8 TB).

#### LED illumination modules and lightguides

The combination of LEDs in LED illumination modules is advisable for multi-wavelength illumination (see below). Liquid light guides eliminate the need for LED placement in close proximity to the mouse’s head; they also improve the illumination angle and radius. Grouping the LEDs simplifies data processing later due to a uniform illumination pattern.

In our system, we combined 530 and 656 nm LEDs with 1-to-7 fibre optic bundle in one module, and 460, 505, and 560 nm LEDs with a single-fibre light guide in another module. Particular components depend on LED wavelength; please see sections ‘The minimum configuration’, ‘LED illumination modules and lightguides’, ‘FAD and Ca2+ measurements’, and ‘Cl- and pH measurements’ for details.

1. Select appropriate beam splitters to combine LEDs for haemodynamic measurements into one assembly and those for fluorescence into another. Arrange the LEDs in the assembly in order of increasing wavelength (the shortest wavelength closer to the output of the optical fibre). Ensure that each LED achieves >95% transmission or reflection.
*Note: Alternatively, use one LED assembly for both haemodynamics and fluorescence (for bright sensors).*
2. Assemble the LED illumination modules: attach collimating lenses (see item 3 in section ‘Optical system’) to the LEDs, place bandpass filters (see item 2 in section ‘Optical system’ and item 2 in section ‘FAD and Ca2+ measurements’) into the designated recesses on the beamsplitter, and affix each LED to the beamsplitter. Connect a lightguide adapter with focusing lens to the beamsplitter’s output.
*Note: Follow the manufacturer’s recommended light direction for the dichroic mirrors. If LED power is suboptimal, use neutral density (ND) filters along with bandpass filters.*
3. Connect a light guide to a lightguide adapter. The selected light guide must be long enough to allow camera movement. For fluorescence, we recommend a 1-metre-long, 3-mm core diameter liquid light guide. For haemodynamics we recommend 1.2 m-long branched light guide (1–7) with 0.6-mm core diameter.
*Note: Handle the light guide carefully to avoid scratching the ends and do not exceed the minimum bend radius. With high-power LEDs, it can be replaced by inexpensive optical fibre.*
4. Adjust the light guide at the proper angle to the plane of the headplate: 30°–60° for haemodynamics, 90° for fluorescence (see item 1 in section ‘Optical system’, item 1 in section ‘LED illumination modules and lightguides’, and item 2 in section ‘FAD and Ca2+ measurements’). It is optimal to adjust the fibre optic bundle for haemodynamics around the lens (see 3D model for the mount in Supplementary File S3) to prevent shadow formation.

#### HbO, HHb, and HbT measurements

HbO and HHb have different absorption spectra (Fig. 5). Two LEDs of wavelengths with distinct molar extinction coefficients are enough to calculate both Δ[HbO] and Δ[HHb] [61].

Our HbO/HHb measuring system includes the core system (see section ‘The minimum configuration’) and the following components: an LED at 656 nm (LCS-0656-07-22, Mightex, CA, USA), a bandpass filter (AT655/30m, Chroma Technology Corp., VT, USA), a beam splitter (LCS-BC25-0605, Mightex, CA, USA), a collimating lens with a lightguide adapter (LGC-019-022-05-V, Mightex, CA, USA) a 1-to-7 fan-out fibre optic bundle (BF76HS01, Thorlabs, NJ, USA), and a custom-made mount for fibre optic bundle (see Supplementary File S3).

1. Select the second LED for haemodynamic imaging based on the haemoglobin absorption spectra (Fig. 5). For intrinsic signal imaging only, we recommend 590 nm and 630 nm. If simultaneous GFP-based protein imaging is used, we recommend 530 nm and 630 nm (Table 2, see also item 1 in section ‘Optical system’).
2. If available, assemble the LED illumination module with a fibre optic bundle and a mount for shadow-free illumination (see section ‘LED illumination modules and lightguides’).
3. Connect the second LED to the LED controller. If you have a 2-channel controller with the proper maximum current, use it for both LEDs.
4. Consider the frame rate (see item 6 in section ‘Optical system’), the number of channels in the multi-channel controller (see item 1 in section ‘Control system’), and the software requirements (see item 3 in section ‘Control system’).

#### FAD and Ca^2+^ measurements

Due to the strong overlap between the excitation and emission spectra of FAD and GCaMP, fluorescent measurements of both Δ[FAD] and Δ[Ca^2+^]_i_ can be performed using the same optical setup. Δ[FAD] can be measured in wild-type mice (e.g. C57BL/6J, IGB RAS, Russia), whereas Δ[Ca^2+^]_i_ can be measured in transgenic animals, e.g. strain GP5.17 (JAX, stock #025393). FAD fluorescence is weak, and FAD concentration changes are of low amplitude, and therefore make a minor (4%–11%) contribution to Ca^2+^ measurements with GCaMP [21].

Fluorescent measurements require simultaneous measurements of Δ[Hb], as dynamically changing Hb absorbs both excitation and emission light [61, 66].

In our setup, fluorescent measurements of FAD and GCaMP are performed using an LED at 470 nm (BLS-LCS-0470-14-22-J, Mightex, CA, USA), a bandpass filter (ET470/24m, Chroma Technology Corp., VT, USA), a BioLED Light Source control module (BLS-3000-2, Mightex, CA, USA), a liquid light guide (#77566 3-mm diameter, 1-m length, Thorlabs, NJ, USA), collimating lenses (LGC-019-022-05-V—2 pcs, EPI-MAO-LG, Mightex, CA, USA), and a fluorescent cube (CUBE-MAO-CARR, Mightex, CA, USA) with filters (59009bs, 59026 m, Chroma Technology Corp., VT, USA).

1. Select an LED of the appropriate excitation wavelength (470 nm for GCaMP, 460 or 470 nm for FAD). Use [67] to determine the optimal wavelength (Table 2). The required LED power depends strongly on the fluorophore brightness, the optical filters’ bandwidth, the camera sensitivity, the exposure time, and the possibilities for temporal binning. In our setup, an output power of 860 mW is suitable for both FAD and GCaMP measurements using spatial binning.
2. Assemble a fluorescence filter cube. Mount a bandpass optical filter facing towards the LED, a dichroic mirror at a 45° angle, and an emission filter facing towards the camera. These components must prevent excitation light from entering the camera while efficiently transmitting light in the emission range. For examples, see [68].
*Note: The cube dimensions determine the filter diameter and, therefore, overall system cost.*
3. Plaсe a collimating lens between the LED (or the light guide, if used; see section ‘Surgery’) and the filter cube to transform the divergent light into a parallel beam.
*Note: An inappropriate lens may create an interference pattern. Processing of such frames is complicated, but possible.*
4. Shield the LED light sources. Seal any gaps through which light could escape to prevent stray light from influencing the recordings.
5. Consider the frame rate (see item 6 in section ‘Optical system’), the number of channels in the multi-channel controller (see item 1 in section ‘Control system’), and the software requirements (see item 3 in section ‘Control system’).

#### Cl- and pH measurements

In transgenic mice expressing ClopHensor [24, 26, 69] ΔpH_i_ with Δ[Cl^-^]_i_ can be measured via fluorescence. The setup includes an LED illumination module with three LEDs for excitation (BLS-LCS-0455-03-22, BLS-LCS-0505-12-22, BLS-LCS-0560-03-22, Mightex, CA, USA), a BioLED Light Source control module (BLS-1000-2, Mightex, CA, USA), bandpass filters (#65-142, Edmund Optics, USA; ET505/20x, ZET561/10x, Chroma Technology Corp., VT, USA), and beam splitters (LCS-BC25-0480, LCS-BC25-0515, Mightex, CA, USA). The other parts remain unchanged, as we select filters for the fluorescence cube as universal for all three fluorophores (FAD, GCaMP6f, and ClopHensor). A 460 nm LED with a narrow bandpass filter is crucial to reach an isosbestic point for pH and therefore to measure Δ[Cl^-^]_i_.

#### Recordings in an awake animal

Anaesthesia significantly affects brain activity, although both the recording procedure and data processing in an awake animal are complicated.

Our setup consists of the Mobile HomeCage^®^ (Neurotar, Finland), a custom-made cage with transparent walls, two C-110 63110 webcams with removed IR filters (Defender, China), and two Helios IR-Plate-2-940 illuminators (Microlight, China). The emission filter (59026 m, Chroma Technology Corp., VT, USA) prevents IR light from entering the main WFOI camera (see section ‘FAD and Ca2+ measurements’ and Fig. 5).

*Note: Based on our experience, the C-110 63110 webcam is not optimal due to its low resolution (640 × 480 pixels) and frame rate (30 fps), which are insufficient for accurately tracking paw and whisker movements in mice. A more suitable configuration would include a resolution of 1280 × 720 pixels and a frame rate of at least 60 fps.*

1. Select an appropriate head fixation system, ensuring that it is safe for the mouse. The fixation system should be comfortable, adjustable in height, quick to secure, and should not interfere with exploratory behaviour.
2. Set up a movable platform under the head fixation system. This could be an air-supported cage, a treadmill, a rotating disk, or a spherical ball.
*Note: An awake animal requires a system that permits running (preferably, in all directions) and grooming without risking head injury. An air-supported cage with opaque walls can create an illusion of free movement; however, we recommend using transparent walls to enable behavioural video recording. See the Discussion section for head fixation and movable platform options.*
3. Mount the necessary components for behavioural video recording, including at least one infrared (IR) illuminator, one IR camera and an IR filter for the main WFOI camera.
*Note: Behaviour significantly affects brain activity (see Fig. 10); therefore, it is essential to segment the recording into active and resting periods. Any spectrum outside the range captured by the brain imaging camera may be used; however, IR light is particularly suitable, as it is invisible to mice and does not introduce an additional sensory stimulus.*
4. Set up additional components, such as an airflow control unit and a pump.
5. Test if the setup enables precise temporal synchronization between the main imaging camera and the behavioural monitoring webcam. We use DeepLabCut [70] to detect both LED illumination start and animal movement.

#### Sensory stimulation

Sensory stimulation in an awake animal is compatible with WFOI. Behaviour can interfere with the brain’s response to stimulus, whereas anaesthesia dramatically reduces brain activation. Sensory stimulation facilitates functional cortical mapping both under physiological conditions (e.g. throughout development) and in disease models (e.g. stroke), and allows for evaluation of the impact of external or experimental factors on fundamental brain functions.

**Figure 6:**
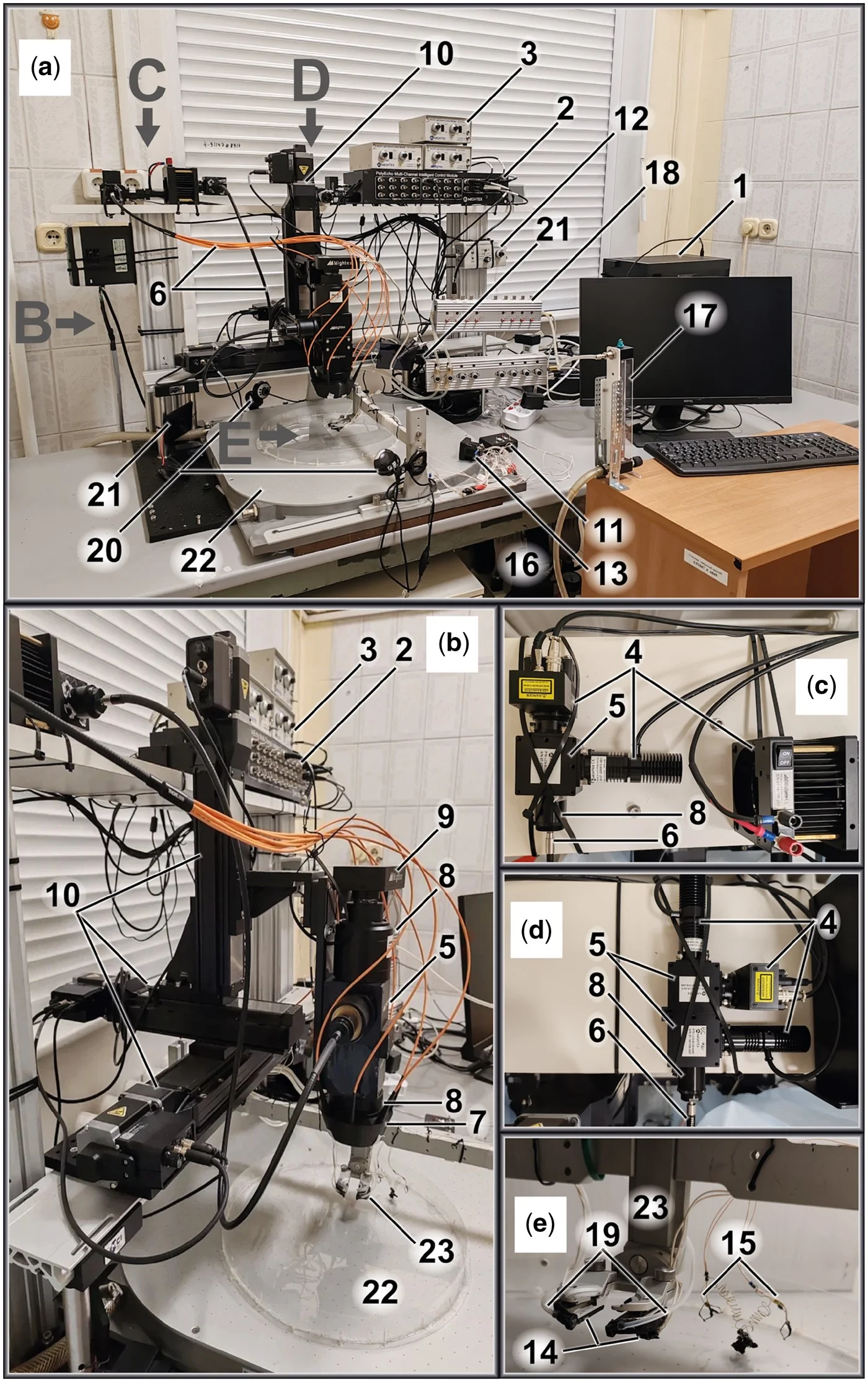
The whole WFOI setup. Panels (**b**), (**c**), (**d**), and (**e**) show more detailed views of the setup in the planes indicated by the arrows in panel (**a**). *Control system*: 1—Computer; 2—PolyEcho; 3—LED controllers. *Brain lighting and recording system*: 4—LEDs with bandpass filters; 5—Cube with filters and dichroics; 6—Fibre optics (1-to-1 and 1-to-7); 7—1-to-7 fibre optic mount; 8—Lenses; 9—CCD camera; 10—XYZ camera positioning. *Eye and limb stimulation*: 11—Left-right switcher; 12—Electrical stimulator; 13—Ammeter; 14—White LED; 15—Hind limb electrodes with tail clip. *Vibrissae stimulation*: 16—Air pump; 17—Rotameter; 18—bio_8_p (air tubes pinching device); 19—Air tube. *Mouse behaviour*: 20—Webcam with extracted IR-filter; 21—IR LEDs. *Mobile HomeCage*: 22—Mobile HomeCage^®^ perforated air-dispensing platform (air pump not shown) with a custom-made cage with transparent walls; 23—Mobile HomeCage^®^ head-fixation apparatus

Our stimulation system for the eyes and hind limbs includes (Fig. 6): an isolated constant current electrical stimulator (Bio-stim2, Biotechnologies, Russia); a microammeter; a 3 mm white LED; a custom-made left-right switcher; and custom-made hind limb electrodes with a tail clip.

Our stimulation system for vibrissae includes: an air pump (607CD22, Thomas Industries Inc., USA); silicone air tubes with diameters of 10, 7, and 2 mm; a rotameter (RWD, China); and a device for pinching air tubes (bio_8_p, Biotechnologies, Russia).

White LEDs and air tubes are fixed on a 3D-printed mount attached to the head fixation frame. We found it optimal for the LED light to be directed downward. Hind limb electrodes are clip-like and are secured to a tail clip with two soft springs. During recording, the electrodes are attached to ring-shaped piercings (two rings per limb, hooked onto the Achilles tendon). The piercings were fabricated from 27G needles; the electrodes were made from segments of a second guitar string; and the tail clip was constructed from a hair claw clip.

In our experiments, we established optimal stimulation parameters that did not elicit active behavioural responses in awake mice, yet reliably evoked distinct local calcium signals in the cortex. For eye stimulation, a current of 100 µA was applied with the LED directed downward (optical power estimated at ∼50–500 μW); for forepaw stimulation, a current of 90 µA was used; and for whisker stimulation, an air flow of 1 L/min. The electrical stimulus was carefully tested and perceived as tactile rather than painful.

### Head-fixation training and imaging procedure

This section describes animal habituation to the head-fixation setup, parameter selection, and the imaging procedure. Since setup configurations and experimental objectives may vary across laboratories, we aim to outline general principles rather than focus on the specifics of our setup.

#### Mouse habituation to head fixation

The habituation protocol acclimatizes the mouse to head fixation in several stages. On day one, the mouse is familiarized with the room for at least 30 min. The mouse is then gently restrained 3*–*5 times by the head in the handler’s hands, then placed on a movable platform near head-fixation apparatus for 1 min. On day two, the same steps are repeated, with the addition of briefly (∼10 s) restraining the mouse by hand next to the head-fixation apparatus. On day three, the animal is again briefly restrained by hand next to the head-fixation apparatus, as on day two, and then head-fixed in the apparatus at least three times for approximately 10 seconds each. On day four, the head fixation for 10 seconds in the apparatus is repeated (at least 3 times), with the addition of a longer head fixation (approximately 1 min) in the apparatus, performed with the lights off and the LEDs on. On the following day, record test resting-state activity for no more than 5 min. The aim is for the mouse to remain calm during fixation, without associating the setup with negative experiences (for the detailed SOP, see Supplementary File S2; for troubleshooting guide, see Supplementary File S4.).

*Notes: Mice should be well acclimatized to handling before starting. Ensure the room’s noise level matches experimental conditions.*

#### Selection of imaging parameters

1. Select parameters according to your experimental goals. Choose a recording duration of 3–5 min for rest-state imaging or longer for stimulation protocols (at least 20 stimuli should be presented at 20-second intervals). Set the frame rate based on signal dynamics: ≥20 Hz for calcium imaging, and 10 Hz for slower signals (Hb, FAD, Cl^-^, and pH). Define the LED sequence appropriate to the indicators used (e.g. LED470 → LED530 → LED470 → LED656 for GCaMP, HbT, and HHb).
2. Configure the imaging system and triggering. Set up the software (e.g. PolyScan4) according to the manufacturer’s instructions, including LED models and controllers. Programme an external TTL trigger matched to the frame rate with pulse duration shorter than the frame interval (e.g. 1 ms), and set the camera exposure time directly in the camera settings so that the pulse duration does not affect it. The number of pulses = frame rate (Hz) × recording duration (s).
3. Set camera and exposure parameters. Use a fixed camera exposure time (e.g. 5 ms) for all recordings to avoid aliasing. Configure the camera to acquire TIFF or RAW images with a bit depth of at least 12 bits (preferably 16 or 32 bits). Set the resolution of 256 × 256 pixels (with binning), with gain set to 0. Enable timestamp-based file naming and continuous preview mode.
4. Calibrate exposure and verify system performance. Place a 1 × 2 cm piece of white paper with blue, green, and red markings in the field of view. Focus the camera, then gradually increase the camera exposure for each LED until glare appears; set the final LED exposure to 80% of the glare threshold. If exposure values differ significantly, reduce intensity of the brightest LED (not below 50% of maximum current, use neutral density filter if needed). Align LED pulse onset with camera triggers, use LED exposure, defined previously. Restore the fixed camera exposure time and enable trigger mode.
  Run a test acquisition to confirm:
  - Even illumination and wavelength-specific disappearance of coloured marks
  - No image shift (≤1 pixel)
  - Absence of interference from other devices (test during activation)
  - Stable ROI intensity traces
  - Zero/non-zero dark signal
*Note: In cases of unstable ROI intensity traces, the global brain-mask mean can be subtracted from each pixel within the same frame. Alternatively, a piece of grey paper can be attached to the visor to facilitate regression of LED intensity from the brain signal (see the Discussion section).*
5. Save your configuration. Archive all camera and system settings to ensure consistent application in future recordings.

#### Imaging procedure

1. Prepare the equipment and protocol. Power on all devices and the control computer. Load the imaging protocol and camera settings. Set the save path and enable automatic file naming.
2. Acclimate the animal. Transfer the mouse to the imaging room and allow 30 min for habituation.
3. Position and prepare the animal. If required, induce and maintain anaesthesia, turn on the heating pad, and apply ophthalmic gel. Fix the mouse securely in the setup, clean the skull, and protect the eyes with a visor (see item 4 in the section ‘Manufacturing headplates’).
*Note: Hand-tightening of the holding screws for mouse fixation may be insufficient.*
4. Set up the imaging system. Focus the camera, turn on one LED in continuous mode, and switch off ambient lighting.
5. Adjust exposure and LED timing. Optimize exposure for each LED to 70%–80% of the glare threshold (see item 4 in section ‘Selection of imaging parameters’). Restore the selected exposure and adjust the LED pulse durations. Switch to trigger mode.
*Note: This procedure must be performed for every animal before each imaging session, as skull transparency can vary significantly between mice and over time.*
6. Start recording and minimize disturbance. Begin imaging. Remain quiet and still to avoid external stimulation. Limit total head-fixation time to 1–2 h.
7. Finish the session and store data. Return the mouse to its home cage and transfer the raw data to external storage.

### Image analysis

Wide-field optical imaging produces sequences of thousands of images. Extracting meaningful signals requires image alignment, noise reduction, and the conversion of pixel intensity into relative changes in haemoglobin concentration or sensor fluorescence. Fluorescent signals must also be corrected for haemoglobin absorption. Additional challenges include automation, faster analysis, and efficient data storage. While various research groups have developed WFOI analysis pipelines in MATLAB and Python, existing open-source scripts lack universality due to variations in hardware, imaging protocols, and analysis methods [71–73]. To address this, we developed a Python-based software package tailored for our WFOI setup, described below and available on GitHub (https://github.com/natalia1896/WIFIOPIA). The key stages of the programme’s operation are listed below.

#### Image annotation and brain masking

Due to manual headplate implantation, the accessible cortical area and the implantation angle may vary slightly across animals. Additional variability may arise from small shifts in objective positioning between imaging sessions. To enable cross-subject comparisons, images are spatially aligned using anatomical landmarks such as bregma and lambda. These landmarks are manually annotated at the start of analysis, and the corresponding rotation and translation parameters are applied to standardize image orientation across sessions. A brain mask is also required to define cortical regions for analysis. This mask can be generated manually or via automated thresholding or neural network-based segmentation. In our workflow, we use manual annotation via a custom graphical interface, though externally generated binary masks are also supported.

#### Image transformation and physiological signal conversion

##### Image transformation

Based on the annotated landmarks, all images are spatially aligned and sorted into 3D arrays (t, w, h), where each array corresponds to a specific excitation wavelength defined by the illumination protocol. Prior to further processing, the dark signal should be subtracted if it is non-zero (I-I_dark_). Signal changes are then computed as relative intensity values (I/I_0_), where I_0_ represents either the mean signal over the full acquisition or over a selected baseline period.

##### Scattering signal conversion to haemoglobin concentrations

For dual-wavelength reflectance recordings, relative changes in haemoglobin concentrations (Δ[HbO], Δ[HHb], Δ[HbT]) are calculated using the modified Beer–Lambert law, as described in [61]:


(1) $${µ}_{a}=-\frac{1}{X(\lambda )}\;*\;\ln\left(\frac{I(\lambda )}{I_{0}(\lambda )}\right),$$

where ${µ}_{a}$ is the absorption coefficient, X(λ) is the wavelength-dependent pathlength, I(λ) is the current pixel intensity, I_0_ (λ) is the baseline intensity for this pixel (the mean signal over the full acquisition or over a selected baseline period). In our experiments, we use wavelength-dependent pathlengths from [61]. Another step is to solve the following system of equations:


(2) $$[\begin{array}{c} \Delta {µ}_{a}(\lambda_{1})\\ \Delta {µ}_{a}(\lambda_{2}) \end{array}]=[\begin{array}{cc} \zeta_{\mathrm{HbO}(\lambda_{1})} & \;\zeta_{\mathrm{HHb}(\lambda_{1})}\\ \zeta_{\mathrm{HbO}(\lambda_{2})} & \zeta_{\mathrm{HHb}(\lambda_{2})} \end{array}]\;*\;[\begin{array}{c} \Delta [\mathrm{HbO}]\\ \Delta [\mathrm{HHb}] \end{array}],$$

where $\zeta_{\mathrm{Hb}(\lambda )}$ is the molar absorption coefficient of specific form of haemoglobin at wavelength λ, Δ[HbO], Δ[HHb] are the changes (relative to baseline) in the molar concentrations of HbO and HHb, respectively, $\Delta {µ}_{a}(\lambda )$ – the changes (relative to baseline) in absorption coefficients.

We use tabulated molar extinction coefficient for haemoglobin in water, as provided by [74]. To calculate $\zeta_{\mathrm{Hb}(\lambda )}$ from the tabulated data, the following equation was used:


(3) $$\zeta_{\mathrm{Hb}(\lambda )}=\ln\left(10\right)*\varepsilon_{\mathrm{Hb}(\lambda )}\approx {2.303*\varepsilon }_{\mathrm{Hb}(\lambda )},$$

where $\zeta_{\mathrm{Hb}(\lambda )}$ is the molar absorption coefficient (in natural logarithm form), and $\varepsilon_{\mathrm{HbO}(\lambda )}$ is the decadic molar absorption coefficient. Both used in the Beer-Lambert law, either in its exponential form ($\frac{I(\lambda )}{I_{0}(\lambda )}=e^{-\zeta *[\mathrm{Hb}]*X(\lambda )}$), or in its absorbance form ($\frac{I(\lambda )}{I_{0}(\lambda )}=10^{-\;\varepsilon *[\mathrm{Hb}]*X(\lambda )}$).

The final solution to the equation is presented below:


(4) $$\Delta [\mathrm{HHb}]=\frac{\left(\zeta_{\mathrm{HbO}(\lambda_{1})}*\;\Delta {µ}_{a}(\lambda_{2})-\;\zeta_{\mathrm{HbO}(\lambda_{2})}*\;\Delta {µ}_{a}(\lambda_{1})\right)}{\left(\zeta_{\mathrm{HbO}(\lambda_{1})}*\;\zeta_{\mathrm{HHb}(\lambda_{2})}-\;\zeta_{\mathrm{HbO}(\lambda_{2})}*\;\zeta_{\mathrm{HbR}(\lambda_{1})}\right)}$$

(5) $$\Delta [\mathrm{HbO}]=\frac{\left(\zeta_{\mathrm{HHb}(\lambda_{1})}*\;\Delta {µ}_{a}(\lambda_{2})-\;\zeta_{\mathrm{HHb}(\lambda_{2})}*\;\Delta {µ}_{a}(\lambda_{1})\right)}{\left(\zeta_{\mathrm{HHb}(\lambda_{1})}*\;\zeta_{\mathrm{HbO}(\lambda_{2})}-\;\zeta_{\mathrm{HHb}(\lambda_{2})}*\;\zeta_{\mathrm{HbO}(\lambda_{1})}\right)}$$

(6) Δ[HbT]= Δ[HbO]+ Δ[HHb]

When only one reflectance wavelength is used, and it lies within the isosbestic range (e.g. 530 nm), changes in ΔI/I_0_ (%) can serve as a proxy for total haemoglobin fluctuations.

##### Correction of fluorescence signal for haemoglobin absorption

At present, the search for optimal fluorescence correction methods remains a methodological challenge in the field. Here, we describe two such approaches.

Our code includes an implementation of haemoglobin correction based on the modified Beer–Lambert model [61]:


(7) $$F_{\mathrm{corr}}=F_{\mathrm{raw}}*e^{\left(\Delta {µ}_{a}(\lambda_{ex})*X_{ex}+\Delta {µ}_{a}(\lambda_{em})*X_{em}\right)},$$

where F_raw_ is the raw fluorescence intensity relative to baseline (F/F_0_), X_*ex*_ and X_*em*_ are photon pathlengths at excitation and emission wavelengths, respectively. However, this approach requires knowledge of the modified excitation and emission photon pathlengths (X*_ex_,* X_*em*_), a parameter that is often experiment-specific and difficult to measure directly [75].

We also implement a data-driven correction using pixel-wise least squares regression [66]. For each pixel (y, x), a linear model is fit to the observed fluorescence F_raw_(t) using the corresponding haemodynamic trace Hb(t) (e.g. Δ[HbT] or ΔI/I_0_) as a predictor:


(8) $$F_{\mathrm{raw}}(t)=\;\beta_{0}+\;\beta_{1}\;*\;Hb(t)+\varepsilon (t),$$

where *β_0_* and *β_1_* are the coefficients, estimated by the linear model, and ε(t) represents the residuals.

The regression is solved using np.linalg.lstsq, minimizing the squared residuals. The corrected fluorescence signal is then represented by the residuals after removing the haemodynamic component:


(9) $$F_{\mathrm{corr}}(t)=\;F_{\mathrm{raw}}(t)-(\beta_{0}+\;\beta_{1}*Hb(t))$$

We compared three correction methods—(1) pixel-wise least-squares regression (LSR), (2) a coefficient-based modified Beer–Lambert model (MBLL) using literature-based coefficients from Ma et al., 2016, and (3) no correction–and selected LSR because it produced faster and cleaner post-stimulus 90% recovery time (median t_90_ = 5.25 s vs. 5.60 s/5.55 s; Kruskal–Wallis H = 15.84, *P* = .00036; Bonferroni-corrected Mann–Whitney *P* = .0008 and .0038) and reduced the post-stimulus undershoot area (area under curve) over 0–2 s (median |PostAUC| = 0.34 for LSR vs. 0.63/0.70; Kruskal–Wallis H = 8.46, *P* = .0146; Bonferroni-corrected Mann–Whitney *P* = .031 and 0.048). The analysis was based on responses to vibrissae stimulation, which provided the most clearly defined hemodynamic signal, using data from three mice (63 stimulus epochs total). Consistent with this choice, Sunil et al. [75] reported pixel-wise variability in optical properties and effective path lengths and used spatial frequency domain imaging (SFDI)-informed Monte-Carlo-derived differential pathlength factors (DPFs) for MBLL correction; lacking SFDI (or equivalent instrumentation) in our setup, we found the data-driven pixel-wise regression to be the most robust and reproducible approach.

The final output includes percentage changes in fluorescence (ΔF/F_0_) and haemoglobin concentrations in µM (Δ[HbO], Δ[HHb], Δ[HbT]), or relative reflectance changes (ΔI/I_0_, %) for single-wavelength recordings. Optionally, to reduce frame-to-frame uniform illumination fluctuations, the global brain-mask mean can be subtracted from each pixel within the same frame. This operation is used solely to suppress a spatially uniform optical component (illumination drift) and is not employed as global signal regression in the sense of functional-connectivity preprocessing. Global-signal methods are debated in the connectivity literature, they may increase specificity to neural correlations by attenuating non-neural fluctuations, but can also alter the correlation structure (including enhancing anticorrelations) [71, 76]. Therefore, when connectivity metrics are analysed, the use of this step should be explicitly stated, and results should be reported both with and without this operation. An alternative approach is regression based on signals from regions of interest whose brightness remains stable throughout the imaging session (e.g. the headplate or visor). However, this method does not eliminate biological noise unrelated to neuronal activity, such as changes in arterial pressure, breath and heart rate, oxygen saturation, or sensor photobleaching. Additional post-processing steps, such as Gaussian smoothing or frequency filtering, can be applied to minimize noise and improve signal quality.

## Results

**Figure 7:**
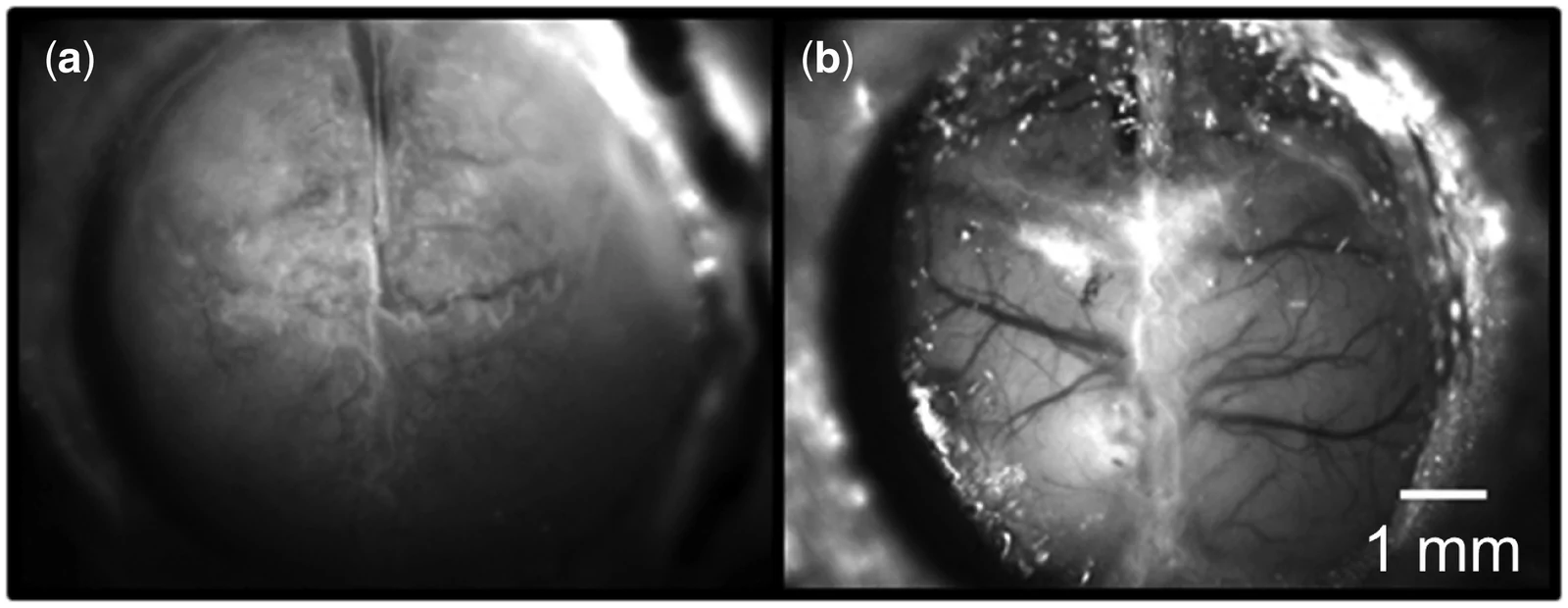
Representative images of the unthinned (**a**) and thinned (**b**) skull at 530 nm in light-scattering (reflectance) mode. During the thinning process, cancellous bone containing diploic vessels are removed

The described protocol for creating a wide cranial window provides an opportunity to accurately measure brain haemodynamics without the need for a craniotomy (Fig. 7). During the thinning process, diploic vessels are removed; therefore, haemodynamics unrelated to neuronal activation does not obscure the underlying activity. Removal of the cancellous bone also improves image quality, as it contributes substantially to light scattering.

**Figure 8:**
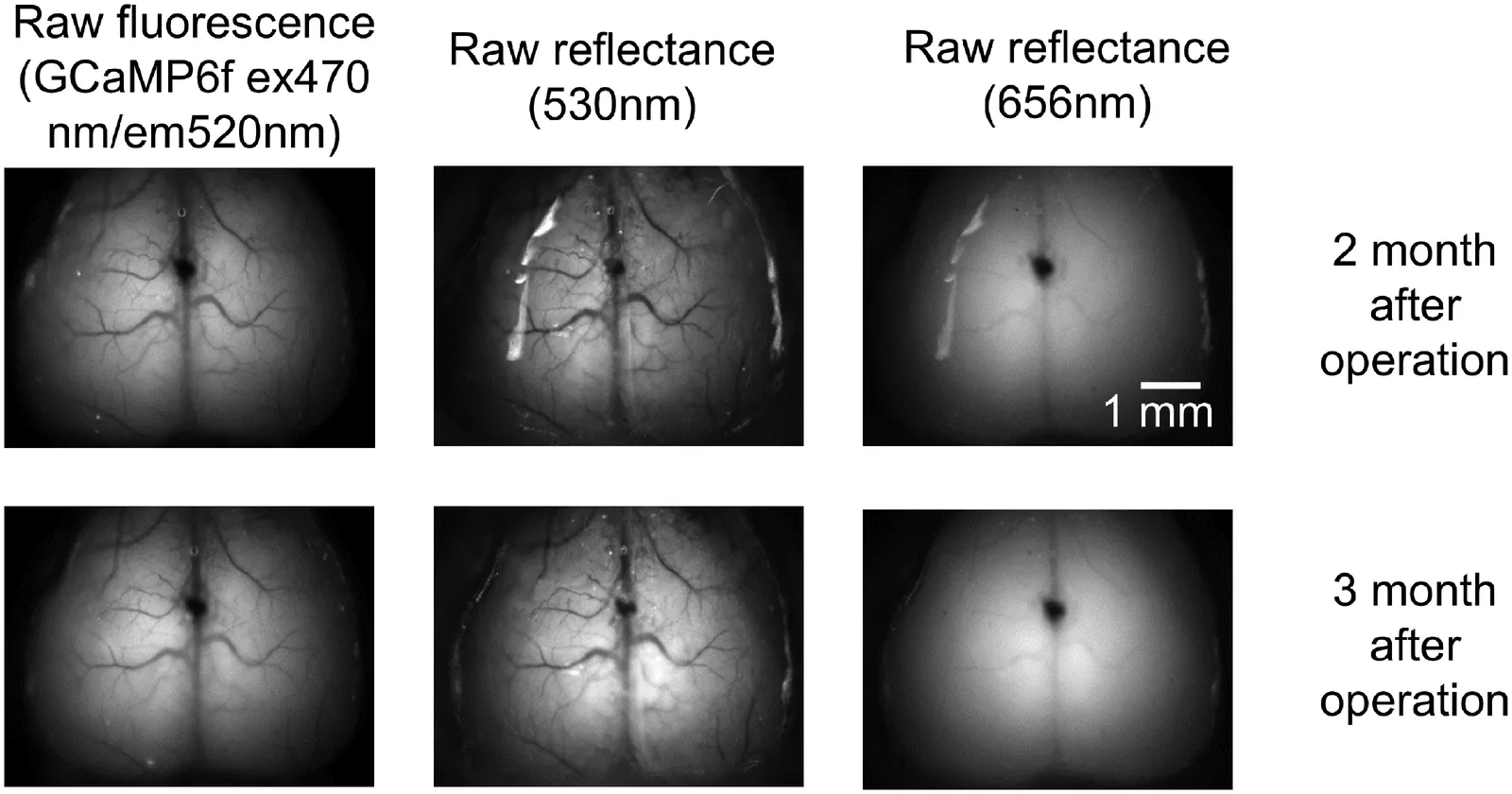
Representative raw images of the cranial window. The proposed protocol for creating a wide cranial window enables stable optical access to the mouse cerebral cortex for over three months. Shown are representative raw fluorescence images from the GCaMP6f sensor and light-scatter images acquired under 530 and 656 nm illumination, taken two and three months after surgery from the same animal

The skull thinning technique provides optical access for recording cortical activity in the same animal for at least three months (Fig. 8). The durability of the cranial implant ensures stable recordings in awake animals during chronic experiments, withstanding multiple fixation and de-clamping procedures.

**Figure 9:**
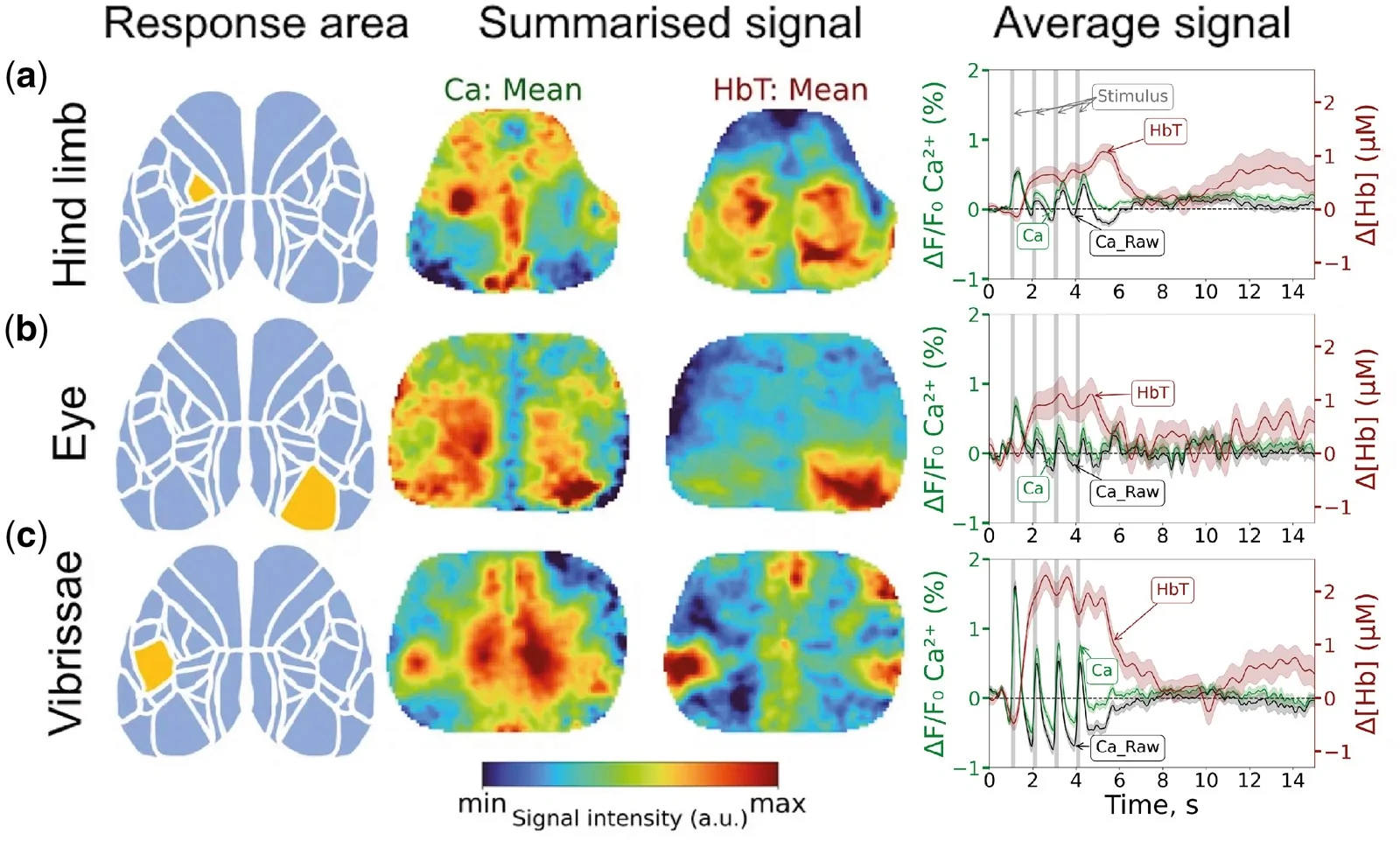
WFOI enables the recording of cortical responses evoked by sensory stimuli of different modalities. Responses to stimulation of the right hind limb (**a**), the left eye (**b**), and the right vibrissae (**c**). From left to right: (1) the location of the corresponding response area according to the anatomical atlas [52]; (2–3) stimulus-locked cumulative heatmaps of the calcium and haemodynamic signals, respectively: calcium integrated over the stimulation epochs and haemodynamics integrated over 0–5 s after stimulus onset to account for slower dynamics; maps are min–max normalized across 21 repeats for an exemplar awake animal selected for clarity and pooled across all stimulus deliveries irrespective of behaviour to more clearly delineate target responsive zones; (4) the average calcium signal from the response area before and after haemodynamic correction (black and green lines, respectively), along with the haemodynamic response (dark red line). For ROI-averaged traces (4), only trials without locomotion were included

The wide cranial window enables the recording of activity across extensive cortical regions in mice, including the motor, somatosensory, parietal, and retrosplenial cortices. To validate the applied brain activity recording and image analysis approaches, we assessed cortical responses to sensory stimulation of different modalities (Fig. 9).

We successfully recorded localized cortical responses to paw, whisker, and eye stimulation. The spatial distribution of activation areas closely matched the anatomically defined sensory regions for each modality, as referenced in standard brain atlases. In awake animals, spontaneous behaviour can broaden the apparent activation, producing non-specific bright areas in the Fig. 9 heatmaps. Stimulation evoked an increase in the calcium signal, followed by a localized haemodynamic response indicative of blood influx.

A stimulation protocol comprising 21 trains of four short, repetitive stimuli (200 ms duration, 1 Hz frequency) was employed. This design enabled precise localization of the response areas.

**Figure 10:**
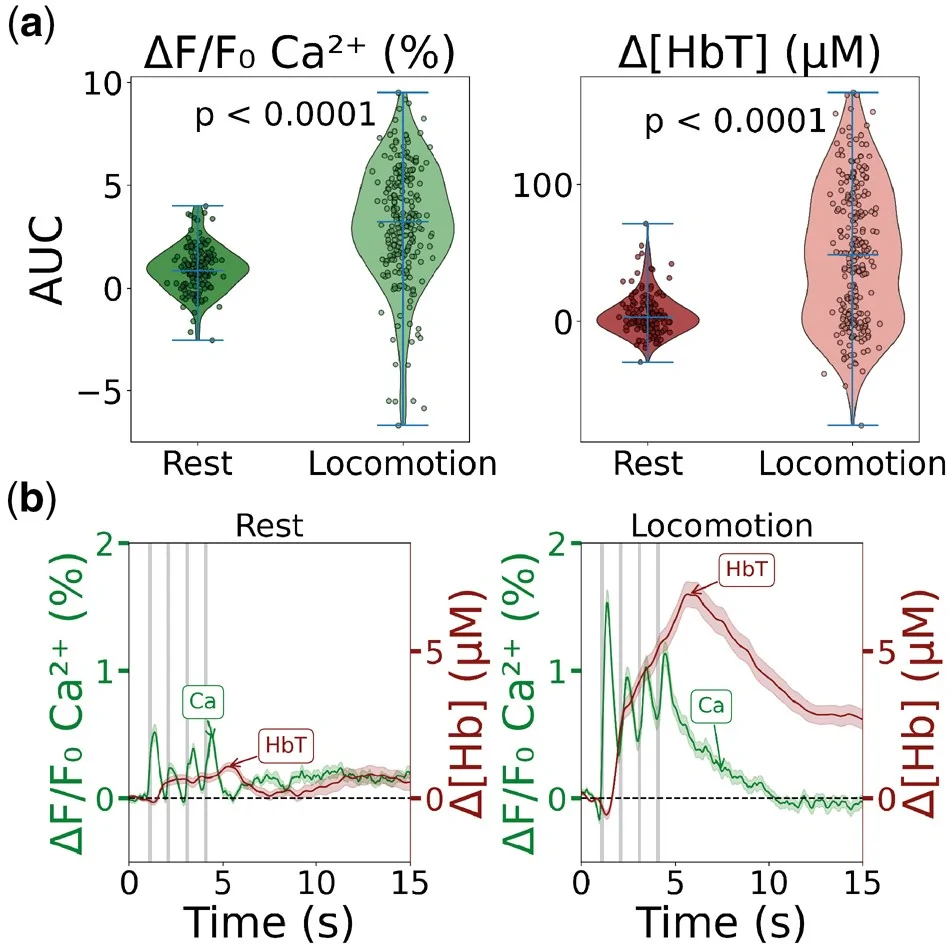
Behaviour significantly affects WFOI responses to stimulation. (**a**) Violin plots showing the area under the curve (AUC) of individual calcium and haemodynamic responses in the somatosensory cortex for hind limb stimulation during calm behaviour (Rest) and active locomotion (Locomotion). (**b**) Responses to hind limb stimulation: averaged Ca^2+^ signal from the response area (green line) and the corresponding haemodynamic response (dark red line), shown for both Rest and Locomotion conditions. Data were collected from 8 mice, with stimulation applied alternately to the left and right hind limbs in separate trials (21 repetitions of 4 pulses with 200 ms duration, 1 Hz frequency, I = 90 μA per limb); responses were recorded from the somatosensory cortex contralateral to the side of stimulation. *P*-values are based on Welch’s *t*-test

Importantly, stimulus-evoked responses in awake animals are affected by behavioural state (Fig. 10). The integral measure of calcium response—area under the curve (AUC)—was significantly lower when animals remained behaviourally calm (0.9 ± 1.11 [mean ± SD], n = 146), compared to trials involving active locomotion [3.16 ± 2.83, n = 232; Welch’s *t*-test: *t*(326.4) = –10.90, *P* < .0001; Cohen’s *d* = –0.97]. A more pronounced effect of movement was observed on the haemodynamic response (measured 0–10 s after stimulation start): AUC of Δ[HbT] was 5.11 ± 15.20 (n = 146) during calm responses and 48.91 ± 50.14 (n = 232) during locomotion [Welch’s *t*-test: *t*(293.4) = –12.43, *P* < .0001; Cohen’s d = –1.08]. Additionally, behavioural activity increased the variability of both calcium and haemodynamic signals, as indicated by Levene’s test (calcium: F = 71.67, *P* < .0001; haemodynamics: F = 173.63, *P* < .0001).

These findings underscore the importance of monitoring behaviour during recordings. In this study, the behavioural state was assessed manually. However, deep learning-based tools, such as DeepLabCut [70], could offer a more objective and reproducible approach for future behavioural classification.

## Discussion

There are two principal approaches to creating cranial windows: transcranial windows, which preserve the skull [10], and craniotomies, which involve skull removal [77]. Two-photon microscopy typically requires either skull thinning [78] or removal with a glass-covered craniotomy [79, 80] on a small (usually 3 in diameter) area of the skull. WFOI, by contrast, benefits from the use of large cranial windows that expose up to 40%–80% of the cortical surface.

While protocols for intrinsic optical signal imaging through the intact skull exist [9, 81, 82], our observations suggest that variability in the diploic vessels may obscure neural signals. Additionally, the intact skull bone significantly reduces signal strength and the signal-to-noise ratio. Removing the trabecular (spongy) bone layer improves optical clarity and can be achieved via skull thinning [21, 36] or skull replacement with transparent materials such as silicone [35], nanocoatings [37], PDMS (polydimethylsiloxane) [40, 83], PET plastic [33], polymethylpentene [84], or glass [34]. Chemical skull-clearing methods have also been developed [85, 86].

Although skull-thinning techniques have been described [21, 36], detailed protocols are often lacking. The method we present is technically simpler than full cranial replacement, carries a lower risk of infection, and minimizes bleeding, when performed with proper skill. A key feature of our protocol is the use of a non-fluorescent, gel-based nail polish as a transparent and protective coating. While this approach is optimized for WFOI (particularly laser contrast imaging), it may also be compatible with two-photon microscopy. Potential limitations include the risk of neuroinflammation, dura mater opacification due to pressure, and long-term bone regrowth. The procedure is time-consuming at first but becomes efficient with practice. We demonstrated that the method is suitable for both recording evoked responses to sensory stimulation and chronic longitudinal imaging. The window remains optically stable and functional for extended periods, enabling repeated within-subject measurements.

Independent of the cranial window technique, stable head fixation is essential for high-quality imaging. This is typically achieved by attaching a fixation device during surgery, which is later mounted in a stereotaxic holder or a mounting frame under the microscope. Devices vary in form: screws, nuts [81], plates [35, 37, 78], or custom clip-based holders [33, 80]. We modified commercial headplates (Neurotar, Finland), originally designed to resemble helicopter blades, to increase cortical access while ensuring firm fixation. These lightweight, compact plates are compatible with the Mobile HomeCage^®^ (Neurotar, Finland).

The WFOI system design varies depending on experimental goals. While commercial systems are available (e.g. Invigilo 3, Neurotar, Finland; OASIS Macro Cortex-Wide Intrinsic Optical Imaging, Mightex, CA, USA), many laboratories build custom setups that support intrinsic optical signal imaging, calcium, or FAD imaging, stroke induction, and other procedures [36, 51, 87], including low-cost configurations [88]. Various solutions for the awake mice *in vivo* imaging exist: rotating disks [40, 44], spheres [39], cylinders [38], treadmills [5, 43, 89], hovercraft platforms [41, 42, 45], and even hammocks [9] or tubes [81]. WFOI is also compatible with virtual reality setups [90].

We developed a flexible and cost-effective WFOI system using commercially available components, which can be readily adapted to various experimental needs. While high-end systems can exceed $200 000, our protocol outlines a minimal configuration suitable for constrained budgets.

We hope that this accessible and adaptable approach will promote the broader use of functional imaging techniques in neuroscience research, contributing to a deeper understanding of brain function and disease mechanisms.

## Supplementary Material

bpaf090_Supplementary_Data

## Data Availability

Supplementary video materials are available on the YouTube channel ‘WIFIOPIA’: https://www.youtube.com/@WIFIOPIA. The code described in this article and the sample data have been deposited in the GitHub https://github.com/natalia1896/WIFIOPIA. Any additional data, if needed, will be provided upon request.
