# Supplementary material for: The protocol for mesoscopic wide-field optical imaging in mice: from zero to hero
Source: Biol Methods Protoc. 2025 Dec 12;11(1):bpaf090. doi: 10.1093/biomethods/bpaf090 (PMC12908863; doi:10.1093/biomethods/bpaf090)
Supplement: bpaf090_Supplementary_Data [file bpaf090_supplementary_data.zip › S4-Troubleshooting.docx]

**Troubleshooting guide for wide-field cranial window surgery and wide-field optical imaging**

| **Problem** | **Possible Cause** | **Solution** |
| --- | --- | --- |
| **Wide-field cranial window surgery** | | |
| Bone regrowth | Incomplete thinning of the growth zone near sutures | Carefully thin the sutures and borders of the frontal bones, polish the sutures, remove fascia from nasal bones. Optionally, remove the germinal layer along the frontal bone edges with a tungsten carbide drill bit. Wait until the cyanoacrylate layer is completely dry; if necessary, accelerate drying with an air flow. |
| Bone cracks | Uneven thinning | Ensure uniform and gradual thinning of all bones in the window area during surgery. Use circular or linear drill movement trough large area. Stabilise working hand. Reverse the rotation direction of the microdrill to prevent widening the crack. When using new drill bits, lightly grind them against sandpaper. |
| Bubbles in coating | Excess gel polish that did not distribute evenly, overly fast brush movements causing foaming | Apply a thin layer of polish, remove excess of polish from brush prior to covering |
| Bleeding from diploic vessels | Diploic vessel damage, mainly near sutures | Minor bleeding usually stops on its own; for severe bleeding, use capillary hemostatic agents or drop of cyanoacrylate glue. Bleeding from diploic vessels is expected; resume thinning once it has stopped |
| Subdural bleeding | Excessive pressure on the skull. Pressure on bone edge. Unstable working hand. Uneven thinning. Microdrill vibrations. Loose head fixation. Bone cracks | Stabilise your working hand and maintain tangential movements. Refine the position of the ear bars. Turn the stereotaxic frame for comfort. Focus the binocular microscope on the thinning site. Do not apply pressure to the thinned surface, as it becomes flexible. Thin through both the frontal and parietal bones along their border. Thin through the large area. Use a high-quality microdrill and drill bits.  Minor bleeding will resolve over time. Speed down the microdrill. Experience is the key factor in achieving consistent results. |
| Loose headplate fixation | Grease / wet / too smooth surface. Gluing to movable tissues. Movement during cyanoacrylate setting. Adapter shape mismatched to strain or age | Glue the headplate to a clean, dry skull surface, avoiding contact with skin, fascia, or muscle. Degrease and dry the headplate before fixation. Use a screw and make small incisions on the nasal bones with a scalpel to roughen the surface and improve adhesion. Adjust the adapter shape with a needle file or prepare adapters suitable for the specific mouse strain and age. Keep your hands steady while the cyanoacrylate sets. Stop any bleeding before applying the resin. Fix the headplate before coating the skull (post-coating attachment is possible but less stable). |
| Difficulties with screw placement | Loose head fixation. Too small or too big indentation | Refine the ear bar position. The indentation should match the diameter of the screw stem and be deep enough for the screw to turn in place. Apply gentle pressure. Cut the thread in the bone using the screw itself. A slight wobble after tightening is acceptable. |
| Dental acrylic resin on cranial window | Overflow during sealing. The gap between the headplate and coating. | Coat the skull with gel polish before applying the resin but after headplate placement. If resin gets on the window, remove it with fine tweezers or a needle moistened with a small amount of acetone after partial polymerisation. Apply resin in small portions, allowing each layer to partially dry before adding the next. |
| Mouse aspiration | Airway obstruction. Fluid aspiration. Respiratory depression by anaesthetic. | Move the tongue gently to the side before positioning the mouse in the stereotaxic frame. Place a small piece of gauze on the nose bones with its edges touching the cotton to allow drainage via capillary action. Reduce isoflurane concentration toward the end of surgery. |
| Eye damage | Contact with irritating substances (alcohol, solvent, depilatory cream), insufficient eye hydration during surgery, excessively bright light | Use ophthalmic gel for eye hydration; reapply as needed. Be careful when applying dental acrylic or other irritants near the eyes. Use foil visor for surgery |
| Intense wound scratching | Inflammatory response in the wound area. Skin irritation due to povidone–iodine (PI) residue. Infection. | Use anti-inflammatory agents. Wipe out PI with 70% ethanol. Ensure the aseptic conditions of all surgical procedures, instruments and solutions. To minimize scratching of unhealed wounds, consider a veterinary collar for no more than 3 days post-surgery. |
| Binocular lenses splashed | Inappropriate drainage. Saline drips on / caudal to the thinning site. | Clean lenses with lens paper and appropriate optical cleaner. Position saline needle rostral to the thinning site. |
| Mouse fur became wet | Head tilted backwards. Improper drainage | Place gauze on the nasal bones with cotton wool for drainage and adjust the head position in the stereotaxic frame |
| Mouse hypothermia | Wet fur. Insufficient heating | Replace wet cotton. Use a warm surgical blanket and a heating pad to maintain body temperature. |
| Equipment rusting | Saline leakage or splashing | Cover equipment with protective film. Use custom-made protective skirts for drill bits. |
| **Wide-field optical imaging** | | |
| Unstable LED brightness | Cross-talk between channels of multi-channel controller. Unstable current supply. LED damage | Set 50% or more of LED intensity (mount ND filters if needed). Use isolated stimulator. Use proper UPS. Normalise the signal to a region of interest outside the brain (e.g., on the headplate). |
| Hemispheres exposed to external light | Insufficient contact between the adapter and the skull, and inadequate light shielding between the stimulating eye LED and the cranial window surface | Check the quality of the adapter. If any openings are present, cover them with dark nail polish. Add an additional light shield if necessary. Direct the stimulating light downward |
| Eye damage or unintended stimulation | Light from brain-illumination LEDs reaching the eyes. Experimenter movements | Use a 3D-printed visor. Sit behind the mouse during recording and remain still throughout imaging |
| Incorrect number of saved frames | Improper camera settings (too long exposure), insufficient memory or processor processing power, unstable cable connections (damaged ports) | Identify the issue, check cable connections, restart the computer |
| Headplate detachment during experiment/training | Loose fixation during surgery, inflammation, excessive force from the experimenter/mouse | If skull damage is minimal and the experimental protocol permits, reattach the headplate (preferably with a screw) and ensure proper postoperative recovery |
| Image displacement | Poor vibration isolation. Mouse movements. Loose mounting screws | Avoid movement during recording. Place the computer and other devices with fans on a separate table. Use an actively vibration-isolated table, a stable head holder, and a rigid camera mount. The mouse platform should prevent the animal from pushing with its limbs. Retighten loosened screws with a screwdriver. Apply alignment algorithms (StackReg [1–3], Template Matching [4,5]), noting that their effectiveness is limited. Binning and Gaussian blurring can partially compensate for vibrations. |
| Mouse anxiety | Poor handling. Distress during the experiment. Injury (e.g., due to improper headplate fixation) | Gradually habituate the mouse to the setup. Place a familiar glove under head-fixation apparatus during fixation. For distressful experimental protocols, perform recordings under anaesthesia (noting that brain activity will be altered). Exclude animals with unmanageable injuries from the experiment. To fasten anxious mouse, wrap it in soft cloth to restrain the body, leaving only the head exposed [6]. |
| Low signal intensity | Low-sensitivity camera. 8-bit images. Dim fluorophore. Low fluorophore expression. Sensor photobleaching | Use spatial and temporal binning. Apply Gaussian smoothing. Switch to a high-sensitivity 16 or 32-bit camera. Consider shorter LED exposure and recording length. Consider a mouse line with higher expression or sensor with low photobleaching (transgenic line, not viral expression, is preferred due to uniform expression throughout brain). |
| Overlap of two LED signals within a single frame | Incorrect LED and camera exposure settings | Verify the recording protocol using a sheet with red, blue, green, and fluorescent marks |
| Shadow on the brain | Illumination from one side only. Adapter or visor too thick | Use shadow-free illumination from multiple directions via branched optical fibres (optic bundle). Employ 3D-printed adapters and visors. |
| Glare | Polarised light entering the camera | Create a flat skull surface using gel polish. Seal one of the branched optical fibre outlets. Use a diffuser or, if necessary, a polarising filter (reduces brightness by 2–4×). |
| Cranial window surface irregularities | Uneven initial coating; later scratching | Minor irregularities do not affect imaging. If they interfere, clean the surface and apply an additional gel polish layer. Alternatively, apply glycerol and a coverslip during imaging. Use a protective cap to prevent scratches. |
| Incorrect settings selected | Experimenter fatigue or inattention | Save configuration presets, use automation scripts (e.g., AutoHotKey [7]), and follow checklists. |

1. Thevenaz, P., Ruttimann, U. E., & Unser, M. (1998). A pyramid approach to subpixel registration based on intensity. IEEE transactions on image processing, 7(1), 27-41.
2. http://bigwww.epfl.ch/publications/
3. http://bigwww.epfl.ch/thevenaz/stackreg/
4. Tseng, Q., Wang, I., Duchemin-Pelletier, E., Azioune, A., Carpi, N., Gao, J., ... & Balland, M. (2011). A new micropatterning method of soft substrates reveals that different tumorigenic signals can promote or reduce cell contraction levels. Lab on a Chip, 11(13), 2231-2240.
5. https://sites.google.com/site/qingzongtseng/template-matching-ij-plugin#h.aj84w9vajptq
6. Kislin, M., Mugantseva, E., Molotkov, D., Kulesskaya, N., Khirug, S., Kirilkin, I., Pryazhnikov, E., Kolikova, J., Toptunov, D., Yuryev, M., Giniatullin, R., Voikar, V., Rivera, C., Rauvala, H., & Khiroug, L. (2014). Flat-floored air-lifted platform: a new method for combining behavior with microscopy or electrophysiology on awake freely moving rodents. Journal of visualized experiments : JoVE, (88), e51869. https://doi.org/10.3791/51869
7. https://www.autohotkey.com/
