# Supplementary material for: The protocol for mesoscopic wide-field optical imaging in mice: from zero to hero
Source: Biol Methods Protoc. 2025 Dec 12;11(1):bpaf090. doi: 10.1093/biomethods/bpaf090 (PMC12908863; doi:10.1093/biomethods/bpaf090)
Supplement: bpaf090_Supplementary_Data [file bpaf090_supplementary_data.zip › bpaf090_Supplementary_Data.zip!/S2-SOP-Headplate-Surgery-Habituation.docx]

**Supplement materials**

# 1 Preparation of adaptor for headplate from epoxy resin

If the shape of the provided adaptor is unsuitable or if 3D-printing is unavailable, custom adapters can be made from epoxy resin.

1. Follow safety precautions: wear gloves and a respirator, and perform all procedures in a fume hood.
2. Make an impression of mouse skull with dental acrylic (e.g. Villacryl S, Zhermack, Poland). Apply dental resin around the intended cranial window layer by layer. For that, use a syringe with a 18G needle.

*It will be more convenient to use shortened 18G needle.*

3. Apply the last layer of the resin, and without allowing it to dry, position the flat metal blank headplate over it.
4. Wait for the resin to completely dry (about 10 minutes) and separate the headplate along with the resin adapter from the mouse's skull. File the existing roughness to achieve a smooth shape of master model.

*Additionally, the sides of master model can be coated with a layer of gel polish to enhance smoothness. Do not cover the underside, as it should remain slightly rough for secure adhesion to the scull.*

5. Make silicone molds from master model. Use a cropped plastic cup, cover its bottom with a thin layer of wax plasticine. Place the master model in the center of the bottom, flat side down. Press it lightly into the plasticine. Mix silicone components due to manufacturer’s instructions (e.g. SF-20A-200, EpoxyMaster, Russia). Add 5 drops of the pigment (e.g. Epic Art Color, Epic Art, Russia). Pour silicone into the prepared plastic cups. Pour the silicone only onto the walls to prevent bubbling. Allow the silicone to cure, which usually takes 8 hours but may vary depending on the manufacturer and proportion of ingredients. Separate the plastic cup. Carefully remove the master model. You can reuse the master model to make more molds.
6. Mix epoxy resin (e.g. 476316047, ArtDealer, Russia). Using a syringe without a needle, measure the required amounts of both components of the epoxy resin in accordance with the manufacturer's instructions (2 ml of mixture per 20 headplate units). Add 1 drop of the black pigment (e.g. Epic Art Color, Epic Art, Russia) and mix them well in an eppendorf tube.

*Take into account the shrinkage of epoxy resin. Due to the poor mixing of two-component polymers (silicone and epoxy resin) near the walls, after the initial mixing, the sample should be transferred to another container and mixed again. The addition of pigment is optional, but it helps to control the completeness of mixing. Try to minimize the presence of bubbles during the process. The Internet provides numerous detailed tutorials on working with epoxy resin and two-component silicone; therefore, this guide describes only the general sequence of steps.*

7. Load the mixture into a clean syringe fitted with a modified (tip-removed) 18G needle. Fill the molds partially and insert metal headplate blanks, avoiding pressure that might introduce bubbles. Allow to cure fully (24–48 h) as per manufacturer’s instructions, then remove the finished headplates.

# 2 Wide-field thinned cranium surgery

**Preparation for surgery. Perform 1 day or more before surgery**

1. Cover all work surfaces, including equipment, with thick plastic wrap to prevent saline from reaching them. Leave the tips of the stereotaxic ear bars, the front parts of the dental clamps, the binocular lenses, the light source, and the saline needle tip unwrapped. Ensure there are no gaps through which saline could leak onto the equipment.

⚠️ *The use of saline is essential to prevent brain overheating caused by drill friction.*

2. Create "skirts" for the drill bits (Fig. S1). To accomplish this, cut out circles with a diameter of approximately 7 millimeters from plastic (~0.5 mm thickness) using scissors, and create a hole in the center that corresponds to the diameter of the leg of the drill bit. Place the "skirt" over the leg and secure it with gel polish approximately 5 millimeters from the bottom of the tip of the drill bit.

*The gel polish should be applied on the side of the tip of the drill bit, such that the saline solution can subsequently flow freely to the edges of the "skirt" and spread apart under the influence of the central force.*

⚠️ *If not, the solution will flow down the leg of the drill bit into the microdrill mechanism.*

⚠️ *If any saline contacts the equipment, immediately dry the area and rinse thoroughly with distilled water to prevent rust formation.*

**Figure S1.** Unused drill bits and used drill bits with protective “skirts.” From left to right: medium-grit diamond drill bit, medium-grit (blue) silicone–diamond polisher, fine-grit (red) silicone–diamond polisher, extra-fine-grit (grey) silicone–diamond polisher, and tungsten carbide drill bit. Note that silicone–diamond polishers lose their sharp tip over time, which makes thinning safer.

3. Dilute the antibiotic Marbofloxacin to a final concentration of 0.00017 mg/mL by adding eleven volumes of water for injection. Prepare aliquots in 1.5 mL microtubes for future use.

*Marbofloxacin may precipitate in saline solutions. All solutions must be prepared aseptically and stored at +4 °C.*

4. Dilute Ketoprophen 30-fold with saline to obtain a final concentration of 1.65 mg/mL. Prepare 1,5 ml aliquots for future use. Ketoprophen is used as an analgesic and anti-inflammatory agent.
5. Dilute the anti-oedema agent Dexamethasone 11.4-fold with saline solution to obtain a final concentration of 0.35 mg/ml. Prepare aliquots for future use.

⚠️ *Different protocols of pre- and post-operative pharmacological treatment are used for cranial window preparation* [1–3]*; we recommend following the guidelines of the local ethical committee and veterinary service.*

6. Prepare 18G cannula tips angled at 45°. Using a needle holder, pinch the tip of the 18-gauge needle and swing it from side to side to break it off, leaving approximately 1.5 cm of the needle. Then, using the needle holder, bend the distal 4 mm of the needle to a 45° angle, taking care not to pinch the lumen.
7. Prepare a 27-gauge L-shaped needle for dispensing glue and removing bubbles from the polish. To do this, use a needle holder to bend the needle 90° along its length, then bend the tip an additional 90° (toward the base) to prevent accidental self-injury during handling. The central section between the two bends should be approximately 0.5 cm. Mount the needle on a syringe or any suitable long handle.
8. Prepare C-shape 27G needle for applying black lacquer markings on the surface of cranial window by breaking off the tip of a 27-gauge needle and bending it into a gentle arc (about a quarter circle).
9. Cut the gauze into strips approximately 2 x 6 cm for scull drainage.
10. Prepare optical paper and lens cleaner for cleaning the binocular lenses.
11. Prepare an adequate supply of cotton swabs, cotton wool and scalpel blades.
12. Make a visor from aluminum foil to protect mouse’s eyes from UV light. Fold the foil four times to obtain a strip approximately 8 × 50 mm in size. Crumple the central part to form a U-shape; the visor should remain nearly flat.

*It would be convenient to keep the listed small supplies in one place.*

13. Extend a silicone tube from the peristaltic pump to the stereotactic device. Attach a blunt medical needle to the distal end of the tube. Secure the needle to a wire or other three-axis movable device, allowing fine adjustment of its position during surgery. Pierce the plastic wrap covering the equipment with the needle tip from the side of the non-dominant hand.
14. Extend the silicone tube from the air pump to the stereotaxis. Fill the tip from the 1000 µl pipette with antibacterial filter material. Attach the tip to the tube.

*If antibacterial filter is unavailable, use sterile cotton wool.*

15. Arrange the peristaltic and air pumps, the heating pad control unit, and the isoflurane system so that their parameters can be easily adjusted during surgery, while ensuring that saline cannot reach them.

**Preparation for surgery. Perform on the day of the operation**

16. Check the availability of all necessary tools and medication (Fig. S2).

**Figure S2.** Instruments and small supplies used during the surgery.

17. Disinfect the workplace with 70% ethanol.
18. Sterilise tools and screws.

⚠️ *Headplates with epoxy resin adapters should not be sterilized using high temperatures or alcohol, as the resin will become soft. Headplates with plastic adapters should not be sterilized using high temperatures. Instead, they can be treated with 70% ethanol prior to gluing.*

19. Prepare a reservoir with distilled water, a nail stick, and cotton swab to wash off the depilatory cream.
20. Prepare a clean cage with fresh water and food. Place 2 food pellets on the floor, so that the mouse can reach them easily until it adapts to the headplate.
21. Rinse the perfusion pump tubing systems (you may turn the pump up to maximum speed):
    a. Peroxide (50 mL).
    b. Distilled water (50 mL).
    c. Fill it with sterile saline.

*Rinsing is not necessary if several procedures are performed in succession.*

22. Setting up the perfusion pump:
    a. Hang the 200 mL saline solution package, lid down. It is important to ensure that there is no risk of leakage of saline solution onto the equipment.
    b. Connect the 18-gauge needle base to the tubing of the perfusion pump and insert the needle into the saline solution container.
    c. Set the flow rate to 1–2 mL/min (while the pump is off). *This can be adjusted to individual preferences.*

*During surgery, we use saline at room temperature. Although lowering the temperature is known to reduce brain damage, prolonged application of cold saline poses a risk of brain hypothermia.*

23. Turn on temperature-controlled pad for animals (37 *°*C).

⚠️ *During surgery, the animal’s fur may become wet, reducing its insulating properties; therefore, proper heating is essential. If bottom heating alone is insufficient, use a warm surgical blanket as additional heat support.*

**Animal preparation**

24. Anaesthetise the animal:
    a. Ensure that the pumps of the isoflurane system are turned on. Confirm that the O₂/air flow to the anaesthesia mask is 1 L/min, both before and after passing through the vaporiser.
    b. Connect the supply line to the induction chamber and set the concentration of isoflurane at 4%.
    c. Place the mouse into the induction chamber for approximately 3 minutes.

*During this time, monitor the animal's breathing rate. When it slows to about one breath per second, remove the mouse from the chamber. If breathing becomes convulsive or irregular, immediately remove the animal to prevent complications.*

25. Gently retract the vibrissae with one finger and shave the fur on the dorsal surface of the head using a razor.

*A thorough shave is unnecessary; removing the majority of the fur from the headplate site is sufficient. If the mouse begins to awaken after shaving, place it back into the induction chamber.*

*Take care not to damage the vibrissae, as this alters brain activity.*

26. Convert isoflurane to 2%.

*Depending on the strain and individual characteristics of the animal, use 1-2.5% isoflurane to maintain anaesthesia. Adjust the concentration to ensure regular breathing at approximately one breath per second while the pedal reflex remains absent.*

27. Secure the mouse within the stereotaxic device.

*Using forceps, gently move the tongue to the one side, than fix the teeth in the holder's opening, slide the mask in place, and secure it. Use the ring or little fingers to move the stereotaxic clamps, being careful not to damage the mouse's skull. Ensure that the clamps rest against the temporal depressions anterior to the ear openings.*

28. Apply eye gel to the mouse's eyes, repeating as necessary to prevent drying (approximately every 15 minutes if saline administration is not used).
29. Lubricate the rectal probe and insert it into the anus of the mouse, securing it with tape around the tail.
30. Administer intramuscular dexamethasone (dose: 0.7 mg/kg; volume: 2 μL per gram body weight). Inject half of the dose into each quadriceps muscle.
31. Subcutaneously inject 0.1 mL of 2% lidocaine under the scalp. Administer antibiotic (Marbofloxacin, D = 8 mg/kg, V = 150 µL/30 g mouse) and analgesic (Ketoprofen, D = 8.5 mg/kg, V = 150 µL/30 g mouse) subcutaneously in the back area (see Notes for 2.2.42 in the main article text).

*Marbofloxacin precipitates in saline, so administer it using a separate syringe.*

32. Place cotton wool beneath and around the mouse's head to absorb any leaking saline solution.

*Keep spare cotton nearby, as it will need to be replaced once it becomes wet.*

33. Adjust the microscope and lighting for binocular viewing.

*Light glares will interfere with the assessment of thinning; therefore, use a lamp with a diffuser. A movable lamp is more convenient.*

*The focus will vary depending on the amount of liquid present and the selected skull area.*

34. Position the stereotaxic frame and the mouse’s head for comfortable thinning: the nose should be lower than bregma, and the frame rotated toward the dominant hand. A standard stereotaxic head position is not required.

**Preparation of the surgical field**

35. Apply depilatory cream to the scalp for 1 minute, covering an area 1–2 mm wider than the planned incision. Avoid contact of the cream with the eyes.

*The application time may be extended to 5 minutes depending on the specific cream lot/formulation, but prolonged contact must be avoided to prevent skin injury.*

36. Remove the cream and fur by scraping them off with the flat end of a wooden nail stick. Wipe away any remaining residue with a cotton swab, then clean the skin with a water-soaked cotton pad.
37. Disinfect gloves with 70% ethanol.
38. Disinfect the mouse’s head by wiping it three times with povidone-iodine, followed by three wipes with 70% ethanol to ensure that no povidone–iodine residue remains, as it may cause skin irritation.

*Ensure that the povidone-iodine and the ethanol do not come into contact with the eyes.*

39. Confirm the absence of a pedal reflex. Check the reflex every 15 minutes to ensure adequate depth of anaesthesia.
40. Excise the scalp using scissors and forceps, ensuring smooth, non-torn edges. The incision should expose the anterior part of the occipital bone, the parietal and frontal bones up to the muscle attachment sites, and 1-2 mm of the nasal bones (up to the anterior eye line).

*The skin will naturally stretch over the occipital region.*

41. To control capillary bleeding, apply haemostatic agent to the wound edges and allow it to dry.
42. Remove the fascia using a cotton swab or scalpel.

*It must be completely cleared from the exposed area, ensuring that no remnants are trapped beneath the implant headplate, as this would compromise fixation. The edges should also be entirely removed to prevent them from becoming entangled by the drill bit during subsequent procedures.*

**Thinning of the skull**

43. Soak the 2 x 6 cm gauze strip (drainage) in saline. Place it over the mouse’s nose so that its edges are in contact with the cotton, while the central part rests on the nasal bones. Use the same gauze to cover and retract the vibrissae downward, as well as to protect the eyes from drying.

*The central portion of the gauze should be in direct contact with the surface of the nasal bones and positioned no closer than the inferior cerebral vein (the boundary between the nasal and frontal bones).*

44. Ensure stable fixation of the mouse's head in the stereotaxic frame: gently test the skull for movement (from side to side) with forceps under the microscope. If necessary, re-secure the ear bars.

*Secure head fixation reduces the risk of brain damage.*

45. Turn on the peristaltic pump at a flow rate of 1–2 mL/min and check that the drainage effectively removes excess fluid.

⚠️ *Without proper drainage, saline may flow down the fur into the mouse’s mouth and nose, increasing the risk of aspiration.*

⚠️ *A continuous flow of saline is required to prevent brain overheating caused by drill friction.*

46. Adjust the position of the peristaltic pump needle so that saline droplets fall rostral to the area being thinned.

*If the droplets fall onto the drill bit or caudally, the bit may disperse the liquid, scatter the liquid and contaminate the binocular microscope lenses.*

47. Set the microdrill speed to approximately 25,000 rpm.

*The speed can be adjusted based on the surgeon’s preference and the mouse’s characteristics (younger mice require lower speeds). The speed may also be reduced when working with the red (soft) silicone-diamond polisher.*

48. Insert the medium-grit diamond drill bit.
49. Rest two fingers of your non-dominant hand on the stereotaxic frame on both sides of the mouse while pulling back its ears. Place the edge of your dominant hand, which holds the drill, on the heating pad, stereotaxic frame, or your other hand.

*This helps stabilize your hand position and prevents damage to the mouse's ears.*

*Adjust the stereotaxic frame position and the tilt of the mouse’s head when thinning different regions of the skull.*

50. Turn on the microdrill.

*Foot pedal-operated models are more convenient.*

51. Use the tip of the drill bit to grind the frontal bones along the perimeter. Bleeding from diploic vessels may occur at this stage; move to a different area and allow it to stop before proceeding.

*The resulting bleeding comes from diploic and emissary vessels, which should be removed during thinning. This bleeding will stop when the vessel is completely removed.*

*However, avoid bleeding from subcortical vessels located beneath the bone tissue.*

*If excessive bleeding occurs, dry the skull with an air stream and apply a Hemostab-soaked cotton swab.*

52. Continue thinning the entire surface of the frontal bones. Maintain tangential motion for uniform thinning.

*The drill angle determines the uniformity of thinning, which is critical for a successful procedure.*

53. Slightly thin the sutures, but avoid full thinning of the anterior ¼ of the outer sagittal sinus, as this may cause severe bleeding. Minimal thinning should be performed in this area (Fig. S3, panel A).

⚠️ *In this region, the external sagittal sinus lies close to the skull surface and is therefore easily damaged, which can disrupt cerebral blood supply. Also take care not to injure the inferior cerebral vein located at the boundary between the nasal and frontal bones. However, the emissary veins branching abundantly from these vessels should be carefully removed.*

54. Turn off the microdrill and peristaltic pump. Turn on the air pump and dry the skull with an air stream. Visually assess the uniformity and degree of skull thinning (Fig. S3, panels D-F).

*A fully thinned skull appears pinkish when dry, brain vasculature is visible through them. Unthinned areas change colour during drying, becoming white and untransparent. The thinned surface may deform under minimal pressure, which can cause brain injury. When thinning the frontal bones, the parietal areas are often affected as well.*

55. Activate the peristaltic pump, keeping in mind the position of the thinned areas. Thin the parietal bones, avoiding touching the thinned areas with medium-grit drill bit. Thinning can be performed using continuous movements from temple to temple along the sagittal suture (Fig. S3, panels B, C). Periodically dry the skull to assess the degree of thinning.

*Uneven thinning (where pinkish thinned regions border white unthinned areas) increases the risk of skull fractures. To prevent this, use continuous circular or linear motions and avoid contacting bones other than the targeted area with the lower part of the drill bit.*

*If a crack forms, change the drill’s rotation direction and avoid touching the damaged site.*

**Figure S3.** Thinning procedure. A: Minimal thinning should be performed in the anterior quarter of the outer sagittal sinus, as this area is prone to severe bleeding. B: Linear motion pattern—thinning can be carried out using continuous movements from temple to temple along the sagittal suture. C: Circular motion pattern. D–F: Indicators of proper thinning on dry (D, E) and wet (F) skull surfaces. Arrows indicate thinned areas (pinkish when dry, with high visibility of brain vasculature), while circles mark unthinned areas (white when dry, with poor transparency).

56. Refine thinning of the sutures and skull surface with medium-grit silicone-diamond polisher (blue). Pay special attention to the boundaries of the frontal bones as they are prone to regrowth (Fig. S4).

*Use the diamond drill bit, followed by a medium (blue) silicone-diamond polisher for removing the outer compact bone layer and most of the spongy bone, leaving the inner compact layer intact.*

⚠️ *Do not use a medium-grit diamond drill bit or medium-grit silicone–diamond polisher on pinkish areas, as this may cause bone cracking.*

**Figure S4.** Bone regrowth typically begins in these areas. Thin and polish them thoroughly to prevent regrowth.

57. Once the skull appears pinkish upon drying, switch to a fine (red) silicone-diamond polisher and perform polishing movements over the entire skull surface.
58. Switch to an extra-fine (gray) silicone-diamond polisher and further polish the entire skull surface. This step is optional.

*The diamond and blue drill bits are effective for thinning the sutures. The red and gray polishers are used for polishing.*

59. Continue thinning until the dry and wet skull appear equally transparent, with clearly visible blood vessels.

⚠️ *A fully thinned skull should not be left dry for extended periods.*

**Application of Coating and Headplate Fixation**

60. Turn off the microdrill and peristaltic pump.
61. Reapply eye gel to the mouse’s eyes.
62. Dry the skull with an air stream. Use the airflow to gently adhere the skin to the skull surface.
63. Apply a small drop of cyanoacrylate glue to the L-shaped needle and carefully spread a thin layer only over the thinned surface. Glue the skin edges to the skull to minimise the risk of infection. Allow the glue to dry completely.

*To speed up drying, an air stream can be used.*

64. Sterilise and test-fit the headplate.
65. Confirm head stability. If necessary, refine ear bars placement.
66. Drill a 1-mm in diameter, ~0.3 mm deep indentation in the occipital bone caudal to the headplate border (in the lower central portion of the occipital bone). Use tungsten carbide drill bit.
67. Insert the M1 screw into a pre-drilled indentation. Use screwdriver and curved forceps to fix it.

*Bones are soft enough to allow screw thread to penetrate it. Continue tightening only as much as necessary to prevent wobbling (it will not be possible to secure it very firmly).*

⚠️ *Do not penetrate the bone completely, as this may trigger cerebellar inflammation.*

68. Use scalpel blade №15 to score nasal bones for proper headplate fixation.
69. Apply glue to the inner rim of the headplate. Gently press the headplate onto the skull and allow the glue to set for 10–15 seconds.

*Apply only light pressure—use the weight of your hands rather than muscle force—to avoid damaging the skull.*

⚠️ *Glue the headplate onto the clean, dry skull surface. Do not glue the headplate onto the skin, fascia or muscles to prevent instability.*

70. Place the aluminum visor over the eyes to protect them from UV exposure.
71. Apply a base coat of gel polish to the entire thinned surface using a brush or an L-shaped needle.

*The layer should be free of bubbles. Add a slightly thicker layer of gel polish along the edges to create a flat surface afterward.*

72. Cure the base coat under a UV lamp for 10 seconds, then pause for 10 seconds, followed by 60 seconds of curing.

*Since the gel polish generates heat during polymerization, pause allows to avoid thermal damage of the brain.*

73. Apply a top coat of gel polish, so that its level reaches the upper edge of the adapter. Try to make surface as flat as possible. Сure under the UV lamp for 10 seconds, then pause for 10 seconds, followed by 60 seconds of curing.

*Application of an excessively thick top coat in one go may cause bubble formation along the edges. To create flat surface load a syringe with 18G needle with gel polish and apply it carefully drop by drop. Use several layers of top coating to create flat surface.*

74. Using the С-shape blunt needle, mark the bregma with black nail polish. Mark lambda and frontal-nasal intersection points if needed.

*Proper thinning renders the bregma visually indistinct.*

75. Prepare the acrylic resin. Use a spatula to place approximately 0.25 g of powder into a silicone mortar. Using an insulin syringe (without a needle), add 200–250 µL of solvent. Mix thoroughly and load the mixture into a syringe. Attach the 18G 45° angled cannula tip to the syringe.

*If necessary, additionally dry the surface around the perimeter with an air stream.*

76. Apply the acrylic resin drop by drop along the outer perimeter of the headplate, allowing each drop to partially set before adding the next.

*This prevents the resin from spreading onto the cranial window or the eyes.*

77. Seal the entire perimeter of the headplate with acrylic resin, covering the skin edges. Apply a thicker layer over the nasal and occipital bones. Avoid any contact of the acrylic resin with the mouse’s eyes.

*Prevent excessive resin buildup on the sides, as it may interfere with proper mounting in the head-fixation frame.*

⚠️ *Bleeding may resume; ensure complete haemostasis, as blood can compromise the solidity of the acrylic.*

*If the resin or nail polish spreads excessively over the cranial window surface, allow it to partially dry and remove the excess with a needle or a small acetone-soaked cotton swab.*

78. After the resin hardens, remove the mouse from the stereotaxic apparatus. Subcutaneously administer 0.5 mL of saline solution.
79. Turn off isoflurane delivery and place the mouse in a clean, heated single-housing cage Monitor the animal closely for at least one-hour post-surgery.
80. For the first three days post-surgery, subcutaneously administer antibiotic (Marbofloxacin, D = 8 mg/kg, V = 150 µL/30 g mouse) and analgesic (Ketoprofen, D = 8.5 mg/kg, V = 150 µL/30 g mouse) daily while monitoring and documenting the animal’s condition, check for signs of inflammation.

⚠️ *Administer injections in the back area near the tail to avoid tension and trauma to the head skin.*

*Well-handled mice should not display aggressive behavior during injections.*

*If the mouse excessively scratches the operation site, consider using a veterinary collar during the first three days after surgery.*

*The rehabilitation period should last at least 14 days, though a one-month recovery is recommended for full restoration*.

# 3 Mouse habituation to head fixation

**Day 1**

1. Turn on the setup. The noise level in the room during habituation should match that during the actual experiment.
2. Transfer the cage with the mouse to the experimental room and allow the animal to acclimate for at least 30 minutes.
3. Handle the mouse and allow it to calm down.

*Note: Mice should be well acclimated to handling beforehand.*

4. Gently restrain the head for 5 seconds. Repeat twice.

*If the mouse resists strongly, release it earlier.*

5. Gently restrain the head for 10 seconds. Repeat three times.
6. Transfer the mouse to the setup and allow it to explore the movable cage for 1 minute.

*Prevent escape attempts with your hand.*

7. Return the mouse to the home cage.

**Day 2**

8. On the following day, repeat steps 1–5, skipping step 4.
9. Adjust the airflow so that a sheet of paper can slide under the cage.
10. Then gently restrain the mouse’s head and bring it close to the head-fixation apparatus. Hold the mouse near the apparatus for 5–10 seconds. Repeat three times.
11. Return the mouse to the home cage.

**Day 3**

12. On the following day, turn on the setup, bring the mouse into the room, and allow 30 minutes for acclimation. Handle the mouse, allow it to calm down, transfer it to the setup, and let it explore for 30 seconds. Gently restrain the head and bring the mouse close to the fixation apparatus for 5–10 seconds.
13. Secure the mouse by the headplate and fix it in the head fixation apparatus. Tighten the screws and release the mouse after 10 seconds. Repeat three times.

*Adjust the height of the fixation apparatus so that the mouse’s head is positioned close to its natural posture.*

14. Return the mouse to the home cage.

**Day 4**

15. On the following day, turn on the setup, bring the mouse into the room, and allow 30 minutes for acclimation.
16. Repeat steps 13.
17. Fix the mouse in the apparatus and lower the camera to the imaging position.
18. Clean the skull with a lint-free tissue moistened with 70% ethanol, cover the eyes with a visor, and turn off the room lights. Turn on LEDs to imitate the condition of the experiment.

*Skull cleaning is unnecessary if protective caps are used.*

19. Wait for 60 seconds.
20. Turn on the light and return the mouse to the home cage.

**Day 5**

21. On the following day, record the resting-state activity for no more than 5 minutes, as described below, and return the mouse to the home cage.

*After this habituation protocol, the mouse should remain calm during head fixation and should not associate the setup with aversive stimuli.*

# 4 Imaging procedure

1. Power on all devices.
2. Turn on the computer.
3. Transfer the cage with the mouse to the experimental room and allow at least 30 minutes for acclimation.
4. Load the imaging protocol and camera settings. Set the file-saving path and enable automatic file naming with a timestamp. An example protocol (Fig. 4) is:
   1) camera: (5 ms on, 20 ms off) × 12,000 repetitions
   2) LED 470 nm: (3 ms on, 47 ms off) × 6,000 repetitions
   3) LED 530 nm: 25 ms off + (5 ms on, 95 ms off) × 3,000 repetitions
   4) LED 656 nm: 75 ms off + (1 ms on, 99 ms off) × 3,000 repetitions

Frame rate: 40 fps. The camera exposure is fixed at 5 ms. LED exposure (LED on-time) must be adjusted individually for each mouse *(see step 15)*. Acquisition frequencies: GCaMP at 470 nm — 20 Hz; haemoglobin absorption at 530 nm — 10 Hz; haemoglobin absorption at 656 nm — 10 Hz. Total recording duration: 5 minutes.

The LED driving current should be set to 50-100 % of the maximum allowable value for each LED. The camera is triggered using a standard TTL signal.

*You may use automation software such as AutoHotkey v2.0 s (Indiania, USA) to minimize human error.*

5. If working distance of your lens is small, raise the camera.
6. If necessary, induce anesthesia using 4% isoflurane and activate the heating pad.
7. Position the mouse in the setup and, if necessary, administer 1.5% isoflurane via a mask.
8. Adjust the height of the head frame so that the head is positioned close to its natural posture. Regulate the airflow of the air platform so that a sheet of paper can slide beneath the platform.
9. Secure all screws with a screwdriver.

*Hand-tightening alone may be insufficient.*

10. Clean the skull with a lint-free tissue moistened with 70% ethanol (or remove the protective cap, if used).
11. Cover the mouse’s eyes with the 3D-printed visor.
12. Set the camera to continuous mode, preview images, and activate an LED in continuous mode.
13. Lower the camera and focus it on the centre of the hemispheres.

*Equal focus across whole hemispheres is not possible due to their curvature.*

14. Turn off the room lights.
15. Adjust the camera exposure to avoid glare formation. Note the exposure time at which glare first appears and write down 70–80% of that value.

*If the 3D-printed mount for 1-7 optic bundle is used, cover some openings with a diffuser (e.g. white medical paper tape) to prevent glare.*

16. Repeat step 15 for all LEDs.
17. Restore the initially selected camera exposure.
18. Adjust LED pulse durations based on the defined values (from step 15).
19. Switch the camera and LEDs to trigger mode.
20. Start imaging.

*Remain behind the mouse during imaging, avoid movement, noise, or changes in lighting, and prevent vibrations. Any sensory stimulus will affect brain activity.*

21. If necessary, record multiple sessions.

*The total duration of head fixation should not exceed two hours, with one hour being optimal.*

22. After imaging, turn on the lights, release the mouse, and return it to the home cage.
23. Transfer the raw data to a secure storage. Keep two copies of the raw data in separate storage locations to prevent data loss.

# Bibliography

1.	Goldey GJ, Roumis DK, Glickfeld LL, Kerlin AM, Reid RC, Bonin V, et al. Removable cranial windows for long-term imaging in awake mice. Nat Protoc. 2014 Nov;9(11):2515–38.

2.	King H, Reiber M, Philippi V, Stirling H, Aulehner K, Bankstahl M, et al. Anesthesia and analgesia for experimental craniotomy in mice and rats: a systematic scoping review comparing the years 2009 and 2019. Front Neurosci. 2023;17:1143109.

3.	Kılıç K, Tang J, Erdener ŞE, Sunil S, Giblin JT, Lee BS, et al. Chronic Imaging of Mouse Brain: From Optical Systems to Functional Ultrasound. Curr Protoc Neurosci. 2020 Sept;93(1):e98.
